# Impact of COVID-19 on myalgic encephalomyelitis/chronic fatigue syndrome-like illness prevalence: A cross-sectional survey

**DOI:** 10.1371/journal.pone.0309810

**Published:** 2024-09-18

**Authors:** Mariah S. Wood, Nicole Halmer, Jeanne Bertolli, Laura B. Amsden, Joshua R. Nugent, Jin-Mann S. Lin, Gretchen Rothrock, Joelle Nadle, Shua J. Chai, Jennifer R. Cope, Jamila H. Champsi, James Yang, Elizabeth R. Unger, Jacek Skarbinski, for STOP-ME/CFS and COVID-SELECT

**Affiliations:** 1 Division of Research, Kaiser Permanente Northern California, Pleasanton, CA, United States of America; 2 Division of High-Consequence Pathogens and Pathology, National Center for Emerging and Zoonotic Infectious Diseases, Centers for Disease Control and Prevention, Atlanta, GA, United States of America; 3 California Emerging Infections Program, Oakland, CA, United States of America; 4 The Permanente Medical Group, Kaiser Permanente Northern California, Oakland, CA, United States of America; 5 Department of Infectious Diseases, South San Francisco Medical Center, Kaiser Permanente Northern California, South San Francisco, CA, United States of America; 6 Department of Adult and Family Medicine, Roseville Riverside Medical Offices, Kaiser Permanente Northern California, Roseville, CA, United States of America; 7 Department of Infectious Diseases, Oakland Medical Center, Kaiser Permanente Northern California, Oakland, CA, United States of America; 8 Physician Researcher Program, Kaiser Permanente Northern California, Oakland, CA, United States of America; Kyung Hee University School of Medicine, REPUBLIC OF KOREA

## Abstract

**Background:**

Myalgic encephalomyelitis/chronic fatigue syndrome (ME/CFS) can be triggered by infectious agents including severe acute respiratory syndrome coronavirus 2 (SARS-CoV-2). However, the impact of the coronavirus disease 2019 (COVID-19) pandemic on ME/CFS prevalence is not well characterized.

**Methods:**

In this population-based cross-sectional study, we enrolled a stratified random sample of 9,825 adult participants in the Kaiser Permanente Northern California (KPNC) integrated health system from July to October 2022 to assess overall ME/CFS-like illness prevalence and the proportion that were identified following COVID-19 illness. We used medical record and survey data to estimate the prevalence of ME/CFS-like illness based on self-reported symptoms congruent with the 2015 Institute of Medicine ME/CFS criteria. History of COVID-19 was based on a positive SARS-CoV-2 nucleic acid amplification test or ICD-10 diagnosis code in the medical record, or self-report of prior COVID-19 on a survey.

**Results:**

Of 2,745,374 adults in the eligible population, an estimated 45,892 (95% confidence interval [CI]: 32,869, 58,914) or 1.67% (CI 1.20%, 2.15%) had ME/CFS-like illness. Among those with ME/CFS-like illness, an estimated 14.12% (CI 3.64%, 24.6%) developed the illness after COVID-19. Among persons who had COVID-19, those with ME/CFS-like illness after COVID-19 were more likely to be unvaccinated and to have had COVID-19 before June 1, 2021. All persons with ME/CFS-like illness had significant impairment in physical, mental, emotional, social, and occupational functioning compared to persons without ME/CFS-like illness.

**Conclusions:**

In a large, integrated health system, 1.67% of adults had ME/CFS-like illness and 14.12% of all persons with ME/CFS-like illness developed it after COVID-19. Though COVID-19 did not substantially increase ME/CFS-like illness in the KPNC population during the study time period, ME/CFS-like illness nevertheless affects a notable portion of this population and is consistent with estimates of ME/CFS prevalence in other populations. Additional attention is needed to improve awareness, diagnosis, and treatment of ME/CFS.

## Introduction

Myalgic encephalomyelitis/chronic fatigue syndrome (ME/CFS) is a multi-system illness that can be profoundly debilitating. It has been recognized for many years under a variety of names and case definitions [1]. In 2015, the Institute of Medicine (IOM; now the National Academies of Medicine) provided a clinical definition of ME/CFS that requires an illness presenting with substantial reduction in the ability to engage in pre-illness activity that lasts for more than six months and is accompanied by new onset fatigue that is not relieved by rest, post-exertional malaise, unrefreshing sleep, and either cognitive impairment or orthostatic intolerance. The IOM definition does not exclude any medical conditions that could explain persistent fatigue [2–4]. The Centers for Disease Control and Prevention (CDC) recommend that clinicians diagnose ME/CFS based on patient self-report of symptoms that match the IOM diagnostic criteria [5]. Viral, bacterial, and parasitic infections have been described as triggers for ME/CFS. While noninfectious triggers are also recognized, ME/CFS is considered a post-acute infection syndrome [6–9]. Post-infectious syndromes have been reported for decades [6, 10]. Reported cases have been both sporadic and associated with outbreaks of known and unknown pathogens [6, 10, 11].

The duration of ME/CFS can span many years [1] implying that the prevalence of ME/CFS will continue to increase with each new wave of triggering infections. However, the additive impact of epidemics of triggering infections on ME/CFS population prevalence has not been comprehensively characterized. Documentation of the “long tail” of ME/CFS following epidemics and the cumulative impact over time is important for effective public health response and preparedness for future epidemics.

Severe acute respiratory syndrome coronavirus 2 (SARS-CoV-2) is the latest emerging pathogen to trigger long-term illness, including new ME/CFS [7, 10, 12, 13]. There is marked interest in understanding the prevalence of post-coronavirus disease 2019 (COVID-19) conditions that persist after acute COVID-19. Given that ME/CFS is one of the possible manifestations of post-COVID-19 conditions and that ME/CFS and post-COVID-19 conditions have strongly overlapping symptoms and biological abnormalities, it is important to understand the prevalence and burden of ME/CFS after COVID-19 [10, 14, 15]. We conducted a population-based cross-sectional study in a large, integrated health system to assess the prevalence of ME/CFS-like illness, estimate the increase in prevalence attributable to the coronavirus disease 2019 (COVID-19) pandemic, and examine the characteristics, symptoms, and functional limitations of persons with ME/CFS-like illness in these groups compared to persons without ME/CFS-like illness, regardless of COVID-19 history.

## Methods

### Setting

Kaiser Permanente Northern California (KPNC) is an integrated health system that serves over four million members and provides comprehensive inpatient and outpatient healthcare in 269 medical offices and 21 hospitals in Northern and Central California [16]. Members receive most clinical services in KPNC facilities and the KPNC member population is similar in sociodemographic characteristics to the overall California population [17].

### Study design, sampling, and recruitment

“Surveillance to Optimize Protocols for Early Identification and Subgrouping of ME/CFS (STOP ME/CFS)” is a population-based cross-sectional study using patient survey (S1 Appendix) and electronic health record (EHR) data to conduct syndromic surveillance of chronic, unexplained fatigue. STOP ME/CFS was expanded in 2022 to include COVID-19 long-term effects through a sub-study, “COVID Standardized Evaluation of Long-term Effects (COVID-SELECT).” This analysis aimed to assess the prevalence of and risk factors for ME/CFS-like illness after COVID-19 and ME/CFS-like illness without prior COVID-19. Epidemiologic and clinical data were obtained from the KPNC Virtual Data Warehouse (Epic, Verona, WI, USA) [18, 19]. The study was approved by the KPNC Institutional Review Board (IRB # 1655522). Informed written consent was obtained for all participants in this study. This activity was reviewed by the CDC and was conducted consistent with applicable federal law and CDC policy.

**Fig 1:**
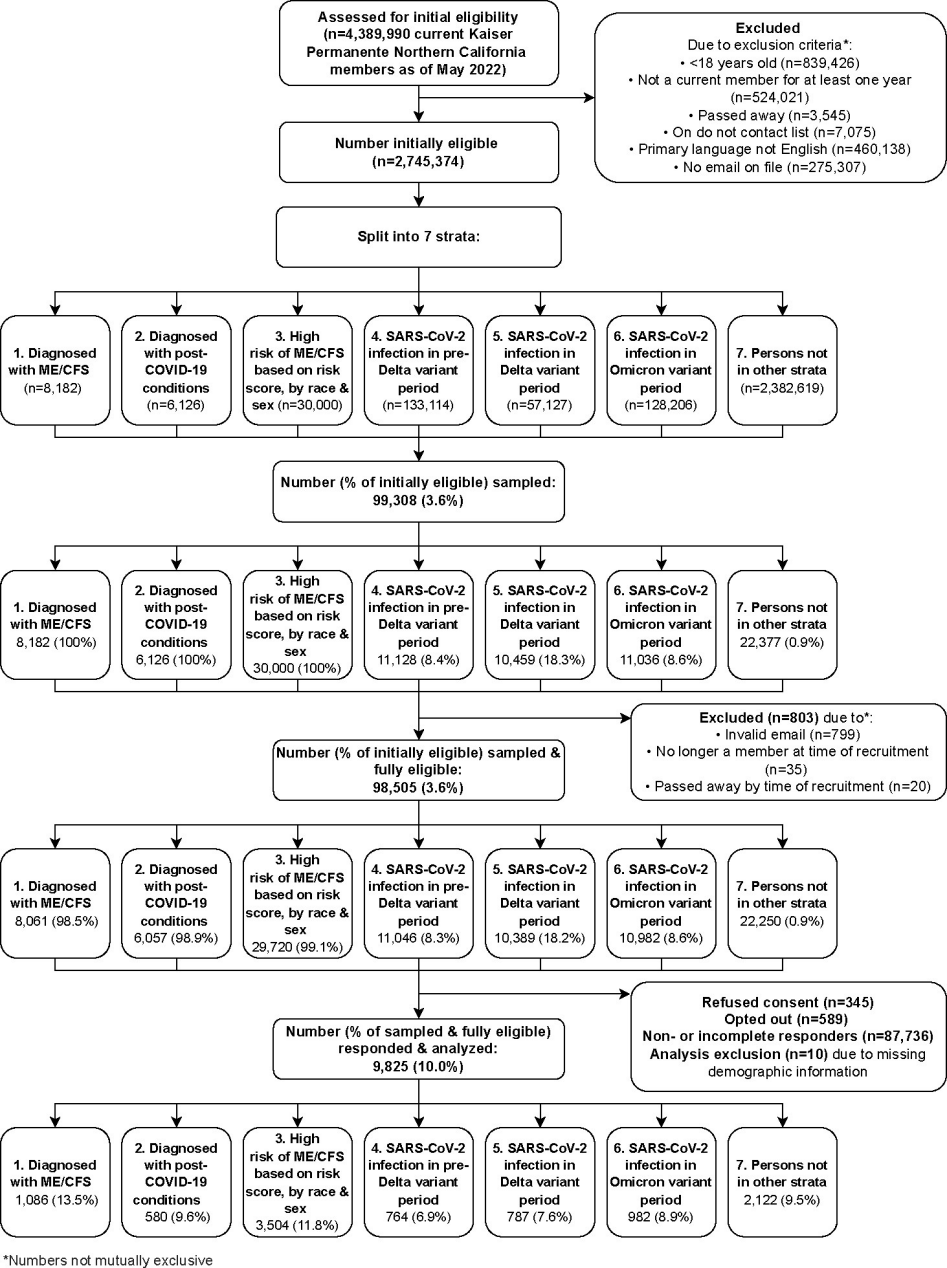
Flow diagram of sampling and recruitment process.

We implemented a stratified sampling design (Fig 1). We developed a sampling frame of persons who met the following inclusion criteria: 1) Current member with KPNC membership for at least one year prior to May 2022; 2) aged ≥18 years; 3) not on KPNC’s do not contact list; 4) working email address on file; 5) English as preferred language for health services. We selected persons for recruitment from seven mutually exclusive, hierarchical strata (once someone qualified for a stratum, in the order presented below, they were no longer eligible for subsequent strata). The first stratum included all persons with an ME/CFS diagnosis in the EHR prior to June 2022 (ICD-10 diagnosis codes: ’R53.82’, ’G93.3’; ICD-09 diagnosis codes: ’323.9’, ’780.71’; number of persons in strata: 8,182). The second stratum included all persons with a diagnosis of post-COVID-19 conditions in the EHR as of May 2022 (ICD-10 diagnosis codes: ’U09.9’, ’B94.8’; number of persons: 6,126).

The third stratum included 30,000 people who may have a higher risk of ME/CFS as defined by IOM criteria but did not have an ME/CFS diagnosis. The probability of having an ME/CFS diagnosis, used as a proxy for the probability of having ME/CFS, was calculated for each current member by training a predictive model on a matched cohort of 6,273 ME/CFS-diagnosed adults and 125,460 reference adults. The cohort consisted of all cases of ME/CFS diagnosis in the EHR from 2007–2020 and controls in a 20:1 ratio who were members the same year their case received their first ME/CFS diagnosis. The model was trained using ME/CFS diagnosis as the outcome and including as covariates age, sex, race/ethnicity, 30 comorbidities from the Elixhauser Comorbidity Index [20], presence of a general fatigue diagnosis in the last year, presence of a fatigue diagnosis prior to one year ago, and 15 health conditions (available upon request) determined by expert co-authors at the CDC. The model was trained on H20.ai [21] using LASSO regression [22], with the penalty term lambda being chosen using ten-fold cross-validation and internally validated using all current eligible KPNC members (Fig 1). Additional model details are provided in S2 Appendix. The trained and validated model was then applied to the current member population excluding persons in strata one or two, and individual probabilities of having an ME/CFS diagnosis were calculated from the model. The stratum was then sub-stratified by sex and race/ethnicity, and the persons with the highest predicted probabilities of ME/CFS diagnosis from each sex-race-ethnicity sub-stratum were used to create stratum three (without this approach this stratum would have lacked representation from people who are not white or not female).

Strata four, five, and six consisted of persons with their first nucleic acid amplification test (NAAT)-confirmed SARS-CoV-2 taking place before the Delta variant was dominant in Northern California (prior to July 5, 2021; number of persons: 133,114), while the Delta variant was dominant (July 5, 2021 to December 14, 2021; number of persons: 57,127) and while the Omicron variant was dominant (December 15, 2021 until time of sampling; number of persons: 128,206), respectively. Stratum seven included all eligible persons not in strata one through six (number of persons: 2,382,619).

To select for people who were more likely to have the relatively rare outcome of ME/CFS, persons were oversampled from strata one to three. In stratum one, 8,061 (98.5%) of 8,182 persons were sampled. In stratum two, 6,057 (98.9%) of 6,126 were sampled. In stratum three, 29,720 (99.1%) of 30,000 were sampled. In strata four through seven, a simple random sample was applied within each stratum. In stratum four, 11,046 (8.3%) of 133,114 were sampled. In stratum five, 10,389 (18.2%) of 57,127 were sampled. In stratum six, 10,982 (8.6%) of 128,206 were sampled. And in stratum seven, 22,250 (0.9%) of 2,382,619 persons were sampled.

All sampled persons were first contacted between July 10, 2022 and October 17, 2022. At first contact, sampled persons were emailed a link to an online survey (S1 Appendix) described as being about general well-being to limit participation bias based on interest in ME/CFS or COVID-19. Three reminder emails and a reminder phone call with a pre-recorded voice message from the principal investigator were sent over the following three weeks. Surveys remained open for 60 days after first contact, meaning the total recruitment period lasted from July 10, 2022 to December 16, 2022. Participants who completed the survey were offered a $10 gift card.

### Exposures, outcomes, and covariates

Our primary exposure is a participant’s first episode of COVID-19 defined as a NAAT-confirmed SARS-CoV-2 infection, a COVID-19 diagnosis in the EHR, or self-report of COVID-19 in the survey. Our primary outcome is timing of a participant’s ME/CFS-like illness onset relative to their first COVID-19 episode. ME/CFS-like illness was determined using a set of survey questions based on the IOM 2015 ME/CFS diagnostic criteria [3]. Using EHR data and survey responses to gather data on COVID-19 and patient reported symptoms, we categorized respondents into three mutually exclusive groups: (1) ME/CFS-like illness after COVID-19 episode; (2) ME/CFS-like illness without prior COVID-19 episode; and (3) No ME/CFS-like illness regardless of COVID-19 history (S1 and S2 Tables). The ME/CFS-like designation is used to indicate that a full clinical evaluation is a requirement for an ME/CFS diagnosis but is not feasible with this survey-only study design.

In addition to defining ME/CFS-like illness status, we defined the temporal relationship between onset of ME/CFS-like illness and COVID-19 (S1 Table). Participants with ME/CFS-like illness after COVID-19 met IOM criteria for ME/CFS-like illness and self-reported onset of fatigue or had a diagnosis of fatigue in the same month or subsequent months after COVID-19. Participants with ME/CFS-like illness without prior COVID-19 met IOM criteria for ME/CFS-like illness and self-reported onset of fatigue or had a diagnosis of fatigue either before COVID-19 or never had COVID-19. For 16 respondents with ME/CFS-like illness for whom we could not define a temporal relationship between fatigue onset and COVID-19 using the process outlined in S1 Table, we conducted detailed chart review using two independent reviewers to assign their analysis group. Of these 16 respondents, 13 were categorized as having ME/CFS-like illness after COVID-19 and three were categorized as having ME/CFS-like illness without prior COVID-19.

Secondary outcomes include the symptoms and functional status of respondents by analysis group as measured by survey components: current symptom inventory, 36-Item Short Form Health Survey (SF-36v2) [23, 24], eight-item Patient Health Questionnaire depression scale (PHQ-8) [25], two-item Generalized Anxiety Disorder scale (GAD-2) [26], Patient-Reported Outcomes Measurement Information System (PROMIS) Short Form Adult v2.0—Cognitive Function 4a (PROMIS SF-CF) [27, 28], and Orthostatic Grading Scale (OGS) [29].

### Statistical analysis

Sampling weights were calculated equal to the inverse of the probability of sample selection. Non-response weights were estimated using Super Learner, an ensemble machine learning method that uses a weighted combination of candidate algorithms to optimize predictive performance via cross-validation [30]. We modeled the probability of survey response within each stratum, adjusting for current age, race/ethnicity, sex, Charlson Comorbidity Index (CCI) score within the last year [31, 32], and body mass index (BMI) within the last two years and generated non-response weights as the inverse of the predicted probability of response (S3 Table). Sampling strata four to seven had higher maximum non-response weights and were trimmed at the 99^th^ percentile. Sampling and non-response weights were multiplied together to determine final survey weights. SF-36 scores and PROMIS cognitive function scores were converted into T-scores [24, 33, 34].

We examined factors associated with ME/CFS-like illness after COVID-19 and with ME/CFS-like illness without prior COVID-19 using modified Poisson regression with robust standard errors [35–37]. Among participants with any history of COVID-19, we assessed the association of ME/CFS-like illness after COVID-19 with age, race/ethnicity, sex, CCI within the last year, BMI within the last year, and COVID-19 vaccination status at time of first COVID-19 infection. Among all participants, we assessed the association between ME/CFS-like illness without prior COVID-19 with age, race/ethnicity, sex, CCI within the last year, and BMI within the last year.

We tested the pairwise associations between each of our three analysis groups (ME/CFS-like illness after COVID-19; ME/CFS-like illness without prior COVID-19; no ME/CFS-like illness) for each of the eight SF-36 functional sub-scales, PHQ-8 and GAD-2 scales, cognitive function scale, and orthostatic grading scale using targeted maximum likelihood estimation (TMLE) [38] with Super Learner [30] adjusting for age, race/ethnicity, sex, CCI, and BMI (S3 Appendix). P-values were adjusted for multiple comparisons using the Benjamini-Hochberg method to control the false discovery rate [39]. Statistical analysis was conducted using R version 4.0.2 [40]. Non-response weights were generated using the SuperLearner package [41], weighted survey data were generated using the survey package [42], and weighted tables were generated using the gtsummary package [43].

## Results

### Prevalence estimates

Of 2,745,374 initially eligible adult members, we sampled 99,308. 98,505 of those sampled were fully eligible, and 9,825 (10.0%) were included in the final analysis. In all, 646 of 9,825 respondents met criteria for ME/CFS-like illness (S4 Table): 77 after COVID-19 and 569 without prior COVID-19. Of 2,745,374 adults in the eligible population, a weighted estimate of 45,892 (95% confidence interval [CI] 32,869, 58,914) people or 1.67% (CI 1.20%, 2.15%) had ME/CFS-like illness. Among those with ME/CFS-like illness, a weighted estimate of 6,480 (CI 1,244, 11,715) people or 14.12% (CI 3.64%, 24.60%) developed the illness after a COVID-19 episode (Table 1).

**Table 1:** Characteristics of persons, stratified by myalgic encephalomyelitis/chronic fatigue syndrome (ME/CFS)-like illness and coronavirus disease 2019 (COVID-19) status in Kaiser Permanente Northern California, 2022.

Characteristic	All		ME/CFS-like illness after COVID-19		ME/CFS-like illness without prior COVID-19		No ME/CFS-like illness^a^	
	n^b^	% (CI)^b^	n^b^	% (CI)^b^	n^b^	% (CI)^b^	n^b^	% (CI)^b^
**Respondents (n)**	9,825		77		569		9,179	
**Estimated population size**	2,745,374	100	6,480 (1,244, 11,715)	0.24 (0.05, 0.43)	39,412 (27,467, 51,357)	1.4 (1, 1.9)	2,699,482 (2,660,356, 2,738,608)	98 (98, 99)
**Any history of COVID-19**	6,000	50 (48, 52)	77	100	317	48 (34, 63)	5,606	50 (48, 52)
**ME/CFS diagnosis in EHR or self-report**	1,280	0.6 (0.48, 0.77)	18	2.1 (0.83, 5.3)	173	5.8 (2.9, 12)	1,089	0.5 (0.41,0.69)
**Post-COVID-19 condition diagnosis in EHR**	693	0.5 (0.35, 0.81)	47	8.6 (3.4, 20)	48	1.3 (0.85, 2.1)	598	0.5 (0.32, 0.78)
**ME/CFS-like illness diagnostic criteria** ^**c**^								
**Fatigue that fits ME/CFS-like illness diagnostic criteria** ^**d**^	3,843	21 (19, 23)	77	100	569	100	3,197	20 (18, 21)
**Fatigue substantially limits ability to pursue work, educational, social, or recreational activities**	4,128	23 (22, 25)	77	100	569	100	3,482	22 (20, 24)
**Does rest make fatigue better?**								
Yes, a lot	2,151	26 (24, 27)	0	0	0	0	2,151	26 (24, 28)
Yes, a little	3,192	26 (24, 28)	26	30 (7.8, 68)	197	46 (32, 61)	2,969	26 (24, 28)
No, not very much	1,957	11 (10, 13)	39	65 (28, 90)	278	45 (31, 60)	1,640	11 (9.6, 12)
No, not at all	403	1.9 (1.4, 2.4)	12	5.1 (1.4, 17)	94	8.6 (3.7, 18)	297	1.8 (1.3, 2.3)
No response	2,122	35 (33, 37)	0	0	0	0	2,122	36 (34, 38)
**Post-exertional malaise that fits ME/CFS-like illness diagnostic criteria** ^**d**^	1,745	5.9 (5.1, 6.9)	77	100	569	100	1,099	4.3 (3.6, 5.2)
**Cognitive impairment that fits ME/CFS-like illness diagnostic criteria** ^**d**^	1,522	7.3 (6.3, 8.3)	74	98 (89, 100)	536	89 (73, 96)	912	5.8 (5.0, 6.9)
**Unrefreshing sleep or problems sleeping that fits ME/CFS-like illness diagnostic criteria** ^**d**^	4,214	28 (26, 29)	77	100	569	100	3,568	26 (25, 28)
**Orthostatic intolerance that fits ME/CFS-like illness diagnostic criteria** ^**d**^	346	1.4 (0.99, 1.9)	18	4.3 (1.4, 13)	156	34 (21, 50)	172	0.9 (0.59, 1.4)
**Years since fatigue onset (mean)**		4 (4.0, 4.8)		2 (1.5, 2.0)		9 (5.4, 12)		4 (3.9, 4.7)
**Age, years (mean)**		50 (49, 50)		36 (33, 39)		45 (40, 50)		50 (49, 50)
**Age, years**								
18–34	1,639	26 (24, 28)	9	54 (19, 85)	86	31 (19, 46)	1,544	26 (24, 27)
35–49	2,372	25 (24, 27)	34	40 (12, 76)	170	22 (13, 36)	2,168	25 (24, 27)
50–64	3,337	24 (22, 26)	26	5.1 (1.8, 14)	241	37 (23, 53)	3,070	24 (22, 26)
65+	2,477	25 (23, 26)	8	1.2 (0.40, 3.7)	72	9.8 (4.1, 22)	2,397	25 (23, 27)
**Race & ethnicity**								
Asian	1,310	22 (20, 24)	3	0.4 (0.09, 1.5)	34	14 (6.4, 29)	1,273	22 (20, 24)
Black	480	5.6 (4.6, 6.7)	5	4.0 (0.65, 21)	29	11 (3.5, 30)	446	5.5 (4.6, 6.6)
Latino/Hispanic	1,595	17 (16, 19)	25	41 (11, 79)	101	27 (15, 43)	1,469	17 (16, 19)
White	5,864	50 (48, 52)	35	53 (18, 85)	348	44 (30, 59)	5,481	50 (48, 52)
Other/unknown	576	5.0 (4.2, 6.0)	9	1.4 (0.47, 4.0)	57	3.5 (1.9, 6.4)	510	5.1 (4.2, 6.0)
**Sex**								
Female	6,680	58 (56, 60)	60	60 (21, 89)	452	75 (57, 86)	6,168	58 (56, 60)
Male	3,145	42 (40, 44)	17	40 (11, 79)	117	25 (14, 43)	3,011	42 (40, 44)
**Body mass index, kg/m** ^**2**^								
< 30	5,158	47 (45, 49)	27	27 (6.3, 67)	252	38 (25, 53)	4,879	48 (46, 50)
≥ 30	3,367	26 (24, 28)	42	40 (11, 79)	272	45 (31, 60)	3,053	26 (24, 27)
**Charlson Comorbidity Index score**								
0 or no visits	5,754	69 (67, 70)	43	91 (74, 97)	301	56 (41, 70)	5,410	69 (67, 71)
1–2	2,868	21 (20, 23)	26	7.7 (2.1, 24)	188	27 (16, 43)	2,654	21 (20, 23)
3+	1,203	10 (9.1, 11)	8	1.4 (0.48, 4.2)	80	17 (8.0, 32)	1,115	10 (9.0, 11)
**Atherosclerotic cardiovascular disease (ASCVD)**	1,739	14 (13, 15)	7	1.1 (0.36, 3.5)	100	16 (8.2, 29)	1,632	14 (13, 15)
**Pulmonary disease**	2,042	14 (12, 15)	21	4.4 (1.6, 11)	157	33 (20, 49)	1,864	13 (12, 15)
**Rheumatologic disease**	251	1.0 (0.72, 1.5)	6	1.4 (0.40, 4.7)	25	0.9 (0.34, 2.3)	220	1.0 (0.71, 1.5)
**Diabetes**	1,223	12 (11, 14)	10	7.1 (1.4, 30)	81	20 (9.8, 38)	1,132	12 (11, 13)
**Renal disease**	583	6.4 (5.5, 7.5)	3	0.5 (0.12, 2.2)	33	10 (3.4, 26)	547	6.4 (5.4, 7.4)
**Cancer**	430	3.8 (3.1, 4.6)	1	0.1 (0.01, 1.0)	22	0.7 (0.27, 1.9)	407	3.8 (3.2, 4.7)
**COVID-19 vaccination status at time of survey**								
None	711	5.4 (4.6, 6.4)	12	24 (4.8, 66)	64	5.3 (2.5, 11)	635	5.4 (4.5, 6.3)
Primary series only	1,266	12 (11, 13)	20	9.3 (2.8, 26)	83	16 (8.6, 28)	1,163	12 (11, 13)
Primary and at least one additional dose	7,666	81 (79, 82)	41	66 (29, 90)	411	78 (66, 87)	7,214	81 (79, 82)
Other	182	1.8 (1.3, 2.4)	4	0.7 (0.17, 2.7)	11	0.5 (0.13, 1.9)	167	1.8 (1.3, 2.5)
**COVID-19 vaccination status at first COVID-19 episode**								
None	1,916	21 (19, 23)	53	96 (87, 99)	117	22 (14, 34)	1,746	21 (19, 22)
Primary series only	1,421	18 (16, 20)	18	3.7 (1.1, 11)	69	40 (22, 60)	1,334	18 (16, 20)
Primary and at least one additional dose	2,323	53 (51, 56)	4	0.5 (0.14, 1.8)	106	36 (19, 58)	2,213	54 (51, 57)
Other	340	7.7 (6.3, 9.4)	2	0.3 (0.05, 1.3)	25	2.4 (0.90, 6.2)	313	7.8 (6.4, 9.6)

EHR = electronic health record; ASCVD = Atherosclerotic cardiovascular disease; CI = 95% confidence interval

^a^Includes all people without ME/CFS-like illness regardless of whether they have had COVID-19

^b^The estimates presented depend on the variable type. The “Respondents” variable presents the unweighted n and unweighted percent. The “Estimated population size” variable presents the weighted n (CI) and weighted percent (CI). Categorical variables present the unweighted n and weighted percent (CI). Continuous variables present the weighted mean (CI)

^c^Meeting the ME/CFS diagnostic criteria is defined as having the following symptoms frequently, at a moderate to severe intensity, for at least six months: fatigue, post-exertional malaise, and unrefreshing sleep or sleep problems, as well as one or both of cognitive impairment or orthostatic intolerance. In addition, the fatigue must substantially limit one’s ability to pursue work, educational, social, or recreational activities, and rest must not make the fatigue substantially better (e.g. not answering “Yes, a lot” to the question “Does rest make fatigue better?”)

^d^Frequent, moderate to severe, lasting ≥ 6 months

### Baseline characteristics

Compared to persons with ME/CFS-like illness without prior COVID-19, persons with ME/CFS-like illness after COVID-19 were younger (mean age 36 vs. 45 years), more likely to be Latino/Hispanic (41% vs. 27%), more likely to have no common comorbidities (CCI score 0 or no visits 91% vs. 56%), and less likely to be female (60% vs. 75%). Persons with ME/CFS-like illness after COVID-19 were more likely to be unvaccinated at time of first COVID-19 illness (no COVID-19 vaccination 96% vs. 22%) compared to persons with ME/CFS-like illness without prior COVID-19 (Table 1). Most (74%) of the persons with ME/CFS-like illness after COVID-19 had their first reported COVID-19 episode prior to June 1, 2021 (S5 Table). Those with ME/CFS-like illness after COVID-19 had experienced fatigue for an average of two years, while those with ME/CFS-like illness without prior COVID-19 had experienced fatigue for an average of nine years (Table 1).

### Symptoms

**Fig 2:**
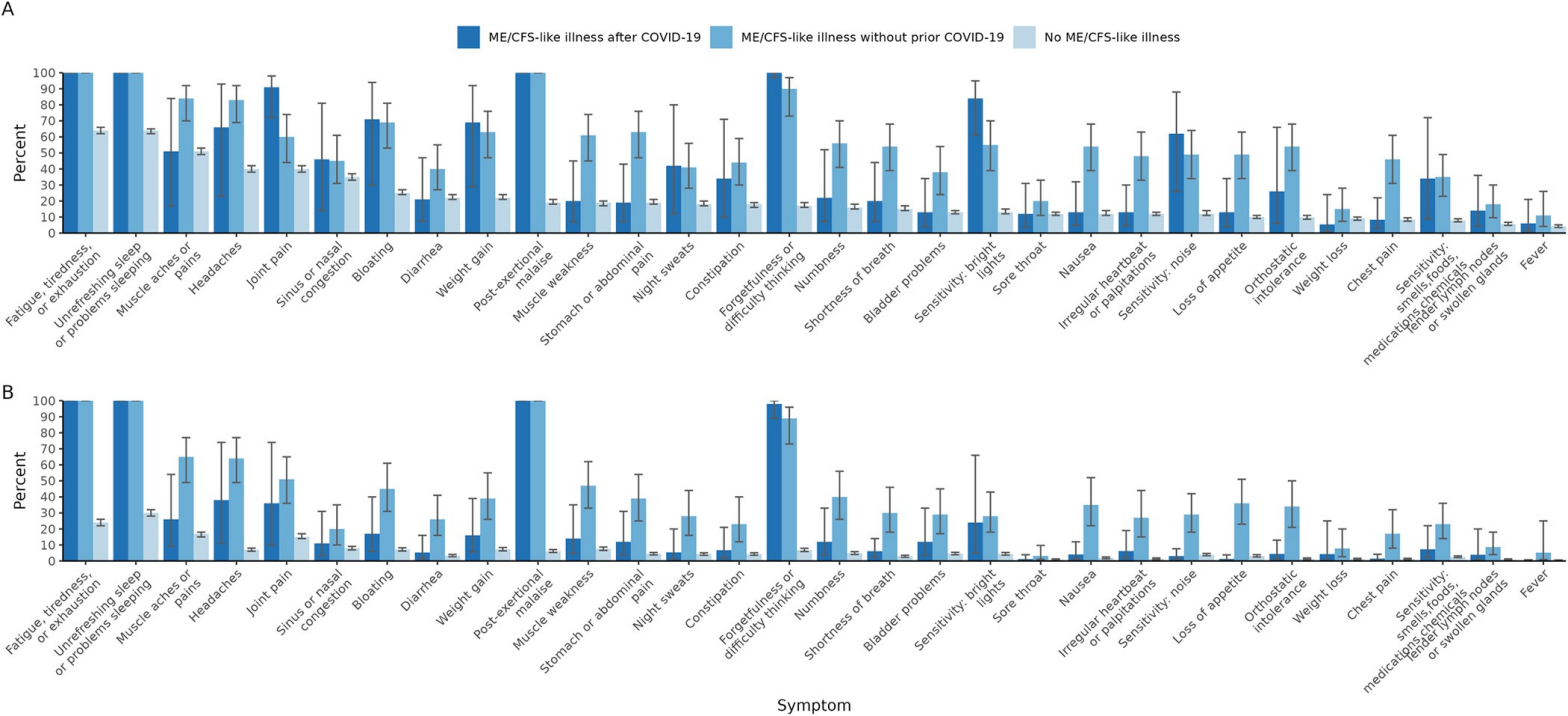
Current symptoms by myalgic encephalomyelitis/chronic fatigue syndrome (ME/CFS)-like illness status. Fig 2 shows the weighted percent of participants in the four weeks prior to survey who: A. report having the included symptoms; B. report having moderate to severe, frequent levels of the included symptoms. The “No ME/CFS Illness” group includes all people without ME/CFS-like illness regardless of whether they have had coronavirus disease 2019 (COVID-19).

Compared to persons with ME/CFS-like illness without prior COVID-19, those with ME/CFS-like illness after COVID-19 generally experienced symptoms at a lower prevalence, as well as at a lower intensity and frequency (Fig 2; S6 and S7 Tables). Besides symptoms in the IOM ME/CFS diagnostic criteria (activity-limiting fatigue, post-exertional malaise, sleep problems, and either cognitive impairment or orthostatic intolerance), all of which were higher in the ME/CFS-like illness groups, both the ME/CFS-like illness after COVID-19 and the ME/CFS-like illness without prior COVID-19 groups experienced 11 out of 25 symptoms at a higher prevalence than the no ME/CFS-like illness group. These included joint pain (91% and 60% vs 40%), sensitivity to bright lights (84% and 55% vs 13%), bloating (71% and 69% vs 25%), weight gain (69% and 63% vs 22%), headaches (66% and 83% vs 40%), sensitivity to noise (62% and 49% vs 12%), sinus or nasal congestion (46% and 45% vs 35%), night sweats (42% and 41% vs 18%), constipation (34% and 44% vs 18%), sensitivity to smells, foods, medications, and chemicals (34% and 35% vs 8%), and tender lymph nodes or swollen glands (14% and 18% vs 6%). Fourteen of 25 symptoms were experienced at a similar prevalence by persons with ME/CFS-like illness after COVID-19 and persons with no ME/CFS-like illness but were more prevalent among those with ME/CFS-like illness without prior COVID-19.

### Factors associated with ME/CFS-like illness

Among persons with any history of COVID-19, older persons (aged ≥ 65 years and 50–64 years versus 18–34 years) were significantly less likely to have ME/CFS-like illness after COVID-19 (adjusted prevalence ratio [aPR] 0.07 [CI 0.02, 0.34] and 0.17 [0.04, 0.75]), as were those identifying as Asian (aPR 0.02 [CI 0, 0.11]). Persons with at least one COVID-19 vaccination at the time of first COVID-19 episode were also significantly less likely to have ME/CFS-like illness after COVID-19 (aPR 0.02 [CI 0.01, 0.05]). Among all persons, older persons (aged ≥65 years versus 18–34 years) were less likely to have ME/CFS without prior COVID-19 (aPR 0.15 [CI 0.05, 0.48]) and persons with a CCI score of three or higher and a CCI score of one to two versus a CCI score of zero were significantly more likely to have ME/CFS-like illness without prior COVID-19 (aPR 4.53 [CI 1.73, 11.87] and 1.87 [1.02, 3.44]) (Table 2).

**Table 2:** Factors associated with myalgic encephalomyelitis/chronic fatigue syndrome (ME/CFS)-like illness after coronavirus disease 2019 (COVID-19) and ME/CFS-like illness without prior COVID-19 in Kaiser Permanente Northern California, 2022.

	ME/CFS-like illness after COVID-19 among persons with any history of COVID-19			ME/CFS-like illness without prior COVID-19 among all persons		
Factor	n (%)^a^	PR^b^ (CI)	aPR^c^ (CI)	n (%)^a^	PR^b^ (CI)	aPR^d^ (CI)
**Total (n)**	**77**	**-**	**-**	**569**	**-**	**-**
**Age, years**						
18–34	9 (0.82)	-^e^	-	86 (1.73)	-	-
35–49	34 (0.62)	0.76 (0.14, 4.02)	0.87 (0.15, 5.09)	170 (1.27)	0.73 (0.34, 1.57)	0.64 (0.29, 1.4)
50–64	26 (0.11)	0.13 (0.03, 0.55)	0.17 (0.04, 0.75)	241 (2.19)	1.26 (0.59, 2.73)	0.89 (0.4, 1.97)
65+	8 (0.04)	0.05 (0.01, 0.2)	0.07 (0.02, 0.34)	72 (0.57)	0.33 (0.12, 0.91)	0.15 (0.05, 0.48)
**Race & ethnicity**						
Asian	3 (0.01)	0.02 (0, 0.08)	0.02 (0, 0.11)	34 (0.94)	0.75 (0.29, 1.9)	0.7 (0.28, 1.77)
Black	5 (0.32)	0.59 (0.08, 4.24)	0.38 (0.04, 3.36)	29 (2.83)	2.24 (0.65, 7.72)	1.23 (0.36, 4.23)
Latino/Hispanic	25 (0.87)	1.62 (0.28, 9.26)	0.81 (0.16, 4.12)	101 (2.24)	1.77 (0.85, 3.69)	1.19 (0.49, 2.89)
White	35 (0.54)	-	-	348 (1.26)	-	-
Other/Unknown	9 (0.11)	0.2 (0.06, 0.75)	0.12 (0.03, 0.49)	57 (1.01)	0.8 (0.4, 1.61)	0.57 (0.27, 1.22)
**Sex**						
Female	60 (0.46)	0.96 (0.17, 5.28)	0.78 (0.07, 8.59)	452 (1.83)	2.08 (0.96, 4.53)	1.85 (0.81, 4.26)
Male	17 (0.49)	-	-	117 (0.88)	-	-
**Charlson Comorbidity Index score**						
0 or no visits	43 (0.59)	-	-	301 (1.17)	-	-
1–2	26 (0.18)	0.31 (0.08, 1.23)	0.41 (0.1, 1.72)	188 (1.83)	1.56 (0.77, 3.18)	1.87 (1.02, 3.44)
3+	8 (0.09)	0.16 (0.05, 0.52)	0.4 (0.09, 1.73)	80 (2.37)	2.02 (0.85, 4.79)	4.53 (1.73, 11.87)
**Body mass index (kg/m** ^**2**^ **)**						
< 30	27 (0.28)	-	-	252 (1.14)	-	-
≥ 30	42 (0.67)	2.44 (0.34, 17.51)	1.71 (0.28, 10.58)	272 (2.5)	2.19 (1.15, 4.17)	1.65 (0.89, 3.08)
**COVID-19 vaccination status at time of first COVID-19 episode**						
None	53 (2.16)	-	-	-	-	-
At least one vaccination	22 (0.03)	0.01 (0, 0.04)	0.02 (0.01, 0.05)	-	-	-

CI = 95% confidence interval

^a^Unweighted row count, weighted row percent

^b^Crude prevalence ratio (CI)

^c^Prevalence ratio (CI) adjusted for age, race/ethnicity, sex, Charlson Comorbidity Index score, BMI, vaccination status at time of first COVID-19 episode

^d^Prevalence ratio (CI) adjusted for age, race/ethnicity, sex, Charlson Comorbidity Index score, BMI

^e^Dash indicates the reference group

### Differences in functional status

We assessed physical, emotional, and social functional status using several instruments (Table 3). Compared to persons with ME/CFS-like illness without prior COVID-19, persons with ME/CFS-like illness after COVID-19 had significantly higher scores (performed better) in the SF-36 physical health domains “role limitations due to physical health” (adjusted mean difference: 6.31 [CI 2.44,10.19]; p-value [p] = 0.002) and “bodily pain” (7.8 [CI 5.2,10.39]; p < 0.001), but had significantly lower scores (performed worse) in the physical health domain “vitality (energy)” (-2.11 [CI -4.02, -0.19], p = 0.04). In addition, persons with ME/CFS-like illness after COVID-19 had significantly better cognitive function scores (1.97 [CI 0.05,3.9], p = 0.05) and less severe orthostatic symptoms (-2.42 [CI -4.36, -0.47]; p = 0.02; negative score indicates better outcome), but significantly higher anxiety scores (0.49 [CI 0.12,0.87]; p-value = 0.01; positive score indicates worse outcome) than persons with ME/CFS-like illness without prior COVID-19. Both persons with ME/CFS-like illness after COVID-19 and ME/CFS-like illness without prior COVID-19 had significantly lower SF-36 scores across all functional domains, lower cognitive function scores, higher orthostatic symptom scores, and higher depression and anxiety scores compared with persons without ME/CFS-like illness. Of persons with ME/CFS-like illness after COVID-19, 2.6% were unable to work due to a disability; of persons with ME/CFS-like illness without prior COVID-19, 26% were unable to work due to a disability.

**Table 3:** Physical, mental, emotional, social, and occupational functioning among persons with myalgic encephalomyelitis/chronic fatigue syndrome (ME/CFS)-like illness after coronavirus disease 2019 (COVID-19), persons with ME/CFS-like illness without prior COVID-19, and persons without ME/CFS-like illness Kaiser Permanente Northern California, 2022.

	1. Total	2. MECFS-like illness after COVID-19	3. ME/CFS-like illness without COVID-19	4. No ME/CFS-like illness^a^	5. ME/CFS-like illness after COVID-19 compared to ME/CFS-like illness without COVID-19		6. ME/CFS-like illness after COVID-19 compared to No ME/CFS-like illness^a^		7. ME/CFS-like illness without COVID-19 compared to No ME/CFS-like illness^a^	
Characteristic	% (CI)^b^	% (CI)^b^	% (CI)^b^	% (CI)^b^	Estimate (CI)^c^	p-value	Estimate (CI)^c^	p-value	Estimate (CI)^c^	p-value
**Total (n)**	9,825	77	569	9,179						
**SF-36 scales (T-score)** ^**d**^ ^,^ ^**i**^										
Physical functioning	49 (49, 50)	45 (37, 54)	38 (33, 43)	50 (49, 50)	1.42 (-2.33, 5.17)	0.49	-6.39 (-8.46, -4.32)	<0.001	-9.89 (-11.09, -8.7)	<0.001
Role-physical	50 (49, 50)	42 (33, 52)	31 (28, 35)	50 (50, 50)	6.31 (2.44, 10.19)	0.002	-8.42 (-10.67, -6.16)	<0.001	-15.81 (-16.62, -15)	<0.001
Bodily pain	51 (50, 51)	45 (37, 53)	34 (31, 37)	51 (50, 51)	7.8 (5.2, 10.39)	<0.001	-6.4 (-8.4, -4.41)	<0.001	-14.34 (-14.91, -13.77)	<0.001
Vitality (energy)	48 (47, 48)	29 (24, 34)	31 (28, 33)	48 (48, 49)	-2.11 (-4.02, -0.19)	0.04	-17.26 (-18.51, -16)	<0.001	-16.06 (-16.59, -15.53)	<0.001
General health	47 (46, 47)	39 (32, 46)	33 (30, 36)	47 (47, 47)	2.48 (-0.58, 5.54)	0.13	-8.52 (-10.31, -6.73)	<0.001	-11.84 (-12.64, -11.05)	<0.001
Social functioning	46 (45, 46)	30 (22, 38)	28 (24, 31)	46 (46, 47)	-0.86 (-3.68, 1.95)	0.56	-14.25 (-16.36, -12.14)	<0.001	-15.99 (-16.76, -15.22)	<0.001
Role-emotional	46 (45, 46)	32 (24, 41)	29 (26, 33)	46 (45, 46)	-0.94 (-5.12, 3.24)	0.66	-11.34 (-13.31, -9.37)	<0.001	-14.14 (-14.92, -13.36)	<0.001
Mental health	47 (46, 47)	34 (26, 42)	30 (26, 34)	47 (46, 47)	3.54 (-0.21, 7.28)	0.08	-9.17 (-11.18, -7.15)	<0.001	-13.25 (-14.12, -12.38)	<0.001
**Change in health from one year ago**										
Better	32 (30, 34)	2.9 (1.0, 8.1)	23 (12, 40)	32 (30, 34)	-	-	-	-	-	-
Same	48 (46, 50)	63 (27, 89)	18 (9.2, 31)	48 (46, 50)	-	-	-	-	-	-
Worse	20 (19, 22)	34 (10, 71)	59 (44, 73)	20 (18, 21)	-	-	-	-	-	-
**PROMIS cognitive function (t-score, mean)** ^**e**^ ^,^ ^**i**^	51 (51, 51)	38 (36, 40)	35 (33, 37)	51 (51, 52)	1.97 (0.05, 3.9)	0.05	-12.34 (-13, -11.67)	<0.001	-15.24 (-15.73, -14.74)	<0.001
**Orthostatic grading scale (0–19, mean)** ^**f**^ ^,^ ^**j**^	5.2 (4.8, 5.6)	6.0 (4.5, 7.5)	9.2 (7.1, 11)	4.8 (4.5, 5.2)	-2.42 (-4.36, -0.47)	0.02	1.29 (0.68, 1.9)	<0.001	4.03 (2.8, 5.25)	<0.001
**PHQ-8 depression score (0–24, mean)** ^**g**^ ^,^ ^**j**^	5.9 (5.7, 6.2)	14.0 (11, 17)	15.8 (14, 18)	5.8 (5.6, 6.0)	-0.93 (-2.53, 0.68)	0.28	6.76 (5.98, 7.53)	<0.001	7.66 (7.15, 8.17)	<0.001
**PHQ-8 categories**										
Depression (≥ 10)	23 (21, 24)	75 (34, 95)	85 (69, 94)	22 (20, 23)	-	-	-	-	-	-
No depression	77 (76, 79)	25 (5.2, 66)	15 (6.4, 31)	78 (77, 80)	-	-	-	-	-	-
**GAD-2 anxiety score (0–6, mean)** ^**h**^ ^,^ ^**j**^	1.51 (1.4, 1.6)	4.68 (3.8, 5.6)	3.83 (3.3, 4.4)	1.47 (1.4, 1.5)	0.49 (0.12, 0.87)	0.01	2.71 (2.5, 2.92)	<0.001	1.72 (1.61, 1.83)	<0.001
**GAD-2 categories**										
None to mild	79 (77, 80)	12 (4.0, 30)	29 (17, 45)	79 (78, 81)	-	-	-	-	-	-
Moderate to severe (≥ 3)	21 (20, 23)	88 (70, 96)	71 (55, 83)	21 (19, 22)	-	-	-	-	-	-
**Work status**										
Unable to work due to health disability	2.6 (2.1, 3.3)	2.6 (1.0, 6.5)	26 (15, 40)	2.3 (1.8, 2.9)	-	-	-	-	-	-
Employed	65 (64, 67)	75 (31, 95)	54 (39, 69)	66 (64, 67)	-	-	-	-	-	-
Not in work force (retired, student, homemaker)	29 (27, 31)	22 (3.5, 70)	9.7 (4.2, 21)	29 (27, 31)	-	-	-	-	-	-
Unemployed	2.8 (2.2, 3.6)	0 (0, 0)	10 (2.8, 30)	2.7 (2.1, 3.5)	-	-	-	-	-	-
Other	0.3 (0.16, 0.66)	0 (0, 0)	0.1 (0.02, 0.96)	0.3 (0.16, 0.67)	-	-	-	-	-	-

CI = 95% confidence interval

^a^Includes all people without ME/CFS-like illness regardless of whether they have had COVID-19

^b^Across groups 1–4, the estimates presented depend on the variable type. Continuous variables present the weighted mean (CI). Categorical variables present the weighted percent (CI)

^c^Across groups 5–7, the estimated mean difference between groups is presented, adjusted for age, race/ethnicity, sex, Charlson Comorbidity Index score, BMI and corrected for multiple testing using the false discovery rate (FDR)

^d^36-Item Short Form Survey v2

^e^PROMIS cognition instrument Adult v2.0—Cognitive Function 4a

^f^5-item Orthostatic Grading Scale

^g^Eight-item Patient Health Questionnaire Depression Scale

^h^Two-item Generalized Anxiety Disorder Scale

^i^For these variables in columns 5, 6, and 7, a positive coefficient indicates a better functional status than the comparison group

^j^For these variables in columns 5, 6, and 7, a positive coefficient indicates a worse functional status than the comparison group

## Discussion

Our study estimates the prevalence of ME/CFS-like illness in the KPNC population and the percentage of ME/CFS-like illness occurring after COVID-19. It assesses factors associated with ME/CFS-like illness and describes the symptoms and physical, mental, emotional, social, and occupational functioning among persons with ME/CFS-like illness in a large, diverse, well-defined population. Those with ME/CFS-like illness after COVID-19 have higher physical functioning and fewer role limitations due to physical health, but experience significantly higher anxiety and significantly less vitality (energy) than those with ME/CFS-like illness without prior COVID-19.

In this study, the weighted point prevalence of ME/CFS-like illness was 1.67% of the total population. Of those with ME/CFS-like illness, 14.12% had the onset after a COVID-19 episode, representing 0.24% of the total patient population. At time of writing there are only a small number of other studies that use IOM criteria to determine ME/CFS after COVID-19, and all of them recruited from non-population-based samples, including Long COVID clinics, COVID-19 hospitalization records, or social media groups [44–47]. All of them assessed prevalence of ME/CFS in a population with post-COVID-19 conditions, rather than prevalence of ME/CFS after COVID-19 in a general population. Populations that have known severe COVID-19 or post-COVID-19 conditions prior to sampling are more likely to have higher prevalence of post-COVID-19 conditions, including ME/CFS, than a general population sample; moreover the different populations being studied make estimates in these studies less directly comparable to our estimates [44–47]. In addition, assessment of IOM criteria is not always clear in these studies; some do not provide measurement details outside of stating they used IOM criteria [46] or suggest that they assessed IOM criteria with a binary questionnaire rather than assessing severity, frequency, and duration of symptoms [3, 4, 47]. Measurement differences add to the difficulty of estimate comparison.

In addition, there are many studies of post-COVID-19 conditions that provide prevalence estimates of core symptoms of ME/CFS among populations with post-COVID-19 conditions that are higher than our ME/CFS-like illness prevalence estimates [48–52]. However, our study required a set of core symptoms based on the IOM diagnostic criteria for ME/CFS [2, 3]. Focusing on a single symptom without considering the severity, frequency, and duration of the other qualifying symptoms does not indicate ME/CFS and does not present a comparable prevalence estimate.

Previous prevalence estimates of ME/CFS (without prior COVID-19), using a variety of inclusion criteria including, in some cases, the IOM criteria, have ranged anywhere from 0.007% to 8.34% in both community-based and clinic-based samples [53–58]. Two prior international meta-analyses found the average prevalence of adult ME/CFS to be 1.45% and between 0.2% to 2.2% [59, 60]. Our prevalence estimate of 1.67% of adults with ME/CFS-like illness is similar to those reported by these studies. Although differences in measurement make direct comparison difficult, our estimate of ME/CFS-like illness in Northern California is supported by prior studies and meta-analyses that have found similar results. This suggests that ME/CFS-like illness in the community may be higher than what is diagnosed in healthcare settings and may affect approximately 1% of the population.

Persons with ME/CFS-like illness after COVID-19 described in this study may offer insights into the early stages of ME/CFS. Those with ME/CFS-like illness after COVID-19 generally had a higher functional status and fewer symptoms compared to those with ME/CFS-like illness without prior COVID-19. This may be related to a shorter duration of symptoms, as those with ME/CFS-like illness after COVID-19 had experienced fatigue for an average of two years (compared to nine years for those with ME/CFS-like illness without prior COVID-19).

Our study suggests that vaccination has a protective effect against ME/CFS-like illness after COVID-19, which is consistent with current literature suggesting that COVID-19 vaccination reduces the risk of post-COVID-19 conditions [7, 13, 61–64]. Regarding the impact of ME/CFS-like illness on daily living, there are some differences between persons with ME/CFS-like illness after COVID-19 and persons with ME/CFS-like illness without prior COVID-19, but ultimately both groups experience much of the same physical and mental symptomology and functional limitations. Both groups experience significantly worse health status compared to those without ME/CFS-like illness, including increased depression and anxiety.

Our analysis has several limitations. First, we only included persons with English listed as their preferred language; 9% of KPNC members prefer a language other than English and were not included in this study. Second, our survey response rate of 10% was lower than ideal, but we conducted rigorous non-response assessment and weighting to adjust for possible non-response bias. Third, we assessed ME/CFS-like illness using the IOM criteria based on self-reported symptom frequency, severity, and duration, not a clinical assessment and diagnosis of ME/CFS. Some persons classified as having ME/CFS-like illness in this study might have an alternative diagnosis that accounts for their symptoms. Fourth, our study has no way to capture asymptomatic SARS-CoV-2 infections and so prevalence of COVID-19 may be underreported. Fifth, our study assumes that ME/CFS-like illness after COVID-19 is most likely related to an episode of COVID-19; however, some people may have developed ME/CFS-like illness after COVID-19 for other reasons. Sixth, we had a small sample size of survey respondents who fit our definition of ME/CFS-like illness after COVID-19 and, as a result, lacked power to critically assess certain associations.

In our large, integrated health system, an estimated 1.67% of persons have ME/CFS-like illness. ME/CFS-like illness has a substantial long-term impact on overall health as persons with ME/CFS-like illness experience many severe symptoms and considerable reductions in physical, mental, emotional, social, and occupational functioning. In our study, 14.12% of those with ME/CFS-like illness developed it after a case of COVID-19, suggesting that the COVID-19 pandemic added to the overall burden of ME/CFS-like illness in our population. COVID-19 vaccination was associated with reduced prevalence of ME/CFS-like illness after COVID-19. Strategies to prevent ME/CFS-like illness and the infections that trigger it, whether COVID-19 or another infection, must be implemented to reduce future cases of ME/CFS [64]. Longitudinal studies and high quality clinical trials are needed to understand the natural history of ME/CFS-like illness both without COVID-19 and after COVID-19 [64], and tools for care and treatment are needed to reduce the morbidity associated with ME/CFS-like illness.

## Supporting information

S1 Table Logic for determining participant grouping. (DOCX)

S2 Table Relationship between exposure, outcome, and primary analysis groups. (DOCX)

S3 Table Design and non-response weights. (DOCX)

S4 Table Participants with ME/CFS-like illness from each of the sampling strata. (DOCX)

S5 Table Characteristics of persons with at least one episode of coronavirus disease 2019 (COVID-19). (DOCX)

S6 Table Reported symptoms in the last 4 weeks. (DOCX)

S7 Table Reported frequent, severe symptoms in the last 4 weeks. (DOCX)

S1 Appendix Survey. (DOCX)

S2 Appendix Predictive model. (DOCX)

S3 Appendix Targeted maximum likelihood estimation description. (DOCX)
