# Supplementary material for: Impact of COVID-19 on myalgic encephalomyelitis/chronic fatigue syndrome-like illness prevalence: A cross-sectional survey
Source: PLoS One. 2024 Sep 18;19(9):e0309810. doi: 10.1371/journal.pone.0309810 (PMC11410243; doi:10.1371/journal.pone.0309810)
Supplement: S1 Table — (DOCX) [file pone.0309810.s001.docx]

| **S1 Table. Logic for determining participant grouping into either myalgic encephalomyelitis/chronic fatigue syndrome (ME/CFS)-like illness after coronavirus disease 2019 (COVID-19), ME/CFS-like illness without prior COVID-19, or No ME/CFS-like illness.** | | | | |
| --- | --- | --- | --- | --- |
| **ME/CFS-like illness group inclusion criteria** | **Information from survey that would fulfill inclusion criteria** | **Information from medical record that would fulfill inclusion criteria** | **Responses that meet inclusion criteria** | |
| Fatigue that substantially limits activities and is not substantially alleviated by rest | - “1. During the past 4 weeks, have you had fatigue, tiredness, or exhaustion?   - **Yes**   - No” - “2. During the past 4 weeks, how often have you had fatigue, tiredness, or exhaustion?   - A little of the time   - Some of the time   - **A good bit of the time**   - **Most of the time**   - **All of the time**” - “3. During the past 4 weeks, how bad was your fatigue, tiredness, or exhaustion?   - Very mild   - Mild   - **Moderate**   - **Severe**   - **Very severe**” - “4. How long have you had fatigue, tiredness, or exhaustion?   - Less than 6 months   - **6 months up to 1 year**   - **1 year up to 3 years**   - **3 years up to 5 years**   - **5 years up to 10 years**   - **10 or more years**” - “5. Has your fatigue substantially limited your ability to pursue your work, educational, social, or recreational activities?   - **Yes**   - No   - Not applicable” - “6. When you experience fatigue, does rest make your fatigue better?   - Yes, a lot   - **Yes, a little**   - **No, not very much**   - **No, not at all**” | - | All of questions 1 through 6 must be answered with one of the responses in bold to meet inclusion criteria for fatigue | |
| Post-exertional malaise | - “7. During the past 4 weeks, have you been unusually fatigued or unwell for at least one day after exerting yourself in any way?   - **Yes**   - No” - “8. During the past 4 weeks, how often have you had unusual fatigue after exertion?   - A little of the time   - Some of the time   - **A good bit of the time**   - **Most of the time**   - **All of the time**” - “9. During the past 4 weeks, how bad was your unusual fatigue after exertion?   - Very mild   - Mild   - **Moderate**   - **Severe**   - **Very severe**” - “10. How long have you had unusual fatigue after exertion?   - Less than 6 months   - **6 months up to 1 year**   - **1 year up to 3 years**   - **3 years up to 5 years**   - **5 years up to 10 years**   - **10 or more years**” | - | All of questions 7 through 10 must be answered with one of the responses in bold to meet inclusion criteria for post-exertional malaise | |
| Unrefreshing sleep or problems sleeping | - “11. During the past 4 weeks, has unrefreshing sleep been a problem for you?   - **Yes**   - No” - “12. During the past 4 weeks, how often have you had unrefreshing sleep?   - A little of the time   - Some of the time   - **A good bit of the time**   - **Most of the time**   - **All of the time**” - “13. During the past 4 weeks, how much of a problem was unrefreshing sleep?   - Very mild   - Mild   - **Moderate**   - **Severe**   - **Very severe**” - “14. How long had you had unrefreshing sleep?   - Less than 6 months   - **6 months up to 1 year**   - **1 year up to 3 years**   - **3 years up to 5 years**   - **5 years up to 10 years**   - **10 or more years**”   OR   - “15. During the past 4 weeks, have you had problems getting to sleep, sleeping through the night, or waking up on time?   - **Yes**   - No” - “16. During the past 4 weeks, how often have you had sleeping problems?   - A little of the time   - Some of the time   - **A good bit of the time**   - **Most of the time**   - **All of the time**” - “17. During the past 4 weeks, how bad were these sleeping problems?   - Very mild   - Mild   - **Moderate**   - **Severe**   - **Very severe**” - “18. How long have you had sleeping problems?   - Less than 6 months   - **6 months up to 1 year**   - **1 year up to 3 years**   - **3 years up to 5 years**   - **5 years up to 10 years**   - **10 or more years**” | - | All of questions 11 through 14 OR all of questions 15 through 18 must be answered with one of the responses in bold to meet inclusion criteria for unrefreshing sleep or problems sleeping | |
| Cognitive impairment | - “19. During the past 4 weeks, have you had forgetfulness or memory problems that caused you to substantially cut back on your activities?   - **Yes**   - No” - “20. During the past 4 weeks, how often have you had forgetfulness or memory problems?   - A little of the time   - Some of the time   - **A good bit of the time**   - **Most of the time**   - **All of the time**” - “21. During the past 4 weeks, how bad were your forgetfulness or memory problems?   - Very mild   - Mild   - **Moderate**   - **Severe**   - **Very severe**” - “22. How long have you had forgetfulness or memory problems?   - Less than 6 months   - **6 months up to 1 year**   - **1 year up to 3 years**   - **3 years up to 5 years**   - **5 years up to 10 years**   - **10 or more years**”   OR   - “23. During the past 4 weeks, have you had difficulty with thinking or concentrating that caused you to substantially cut back on your activities?   - **Yes**   - No” - “24. During the past 4 weeks, how often have you had difficulty with thinking or concentrating?   - A little of the time   - Some of the time   - **A good bit of the time**   - **Most of the time**   - **All of the time**” - “25. During the past 4 weeks, how severe was your difficulty with thinking or concentrating?   - Very mild   - Mild   - **Moderate**   - **Severe**   - **Very severe**” - “26. How long have you had difficulty with thinking or concentrating?   - Less than 6 months   - **6 months up to 1 year**   - **1 year up to 3 years**   - **3 years up to 5 years**   - **5 years up to 10 years**   - **10 or more years**” | - | All of questions 19 through 22 OR all of questions 23 through 26 must be answered with one of the responses in bold to meet inclusion criteria for cognitive impairment | |
| Orthostatic intolerance | - “27. During the past 4 weeks, have you had dizziness or fainting problems?   - **Yes**   - No” - “28. During the past 4 weeks, how often have you had dizziness or fainting problems?   - A little of the time   - Some of the time   - **A good bit of the time**   - **Most of the time**   - **All of the time**” - “29. During the past 4 weeks, how bad was your dizziness or fainting problems?   - Very mild   - Mild   - **Moderate**   - **Severe**   - **Very severe**” - “30. How long have you had dizziness or fainting problems?   - Less than 6 months   - **6 months up to 1 year**   - **1 year up to 3 years**   - **3 years up to 5 years**   - **5 years up to 10 years**   - **10 or more years”** | - | All of questions 27 through 30 must be answered with one of the responses in bold to meet inclusion criteria for orthostatic intolerance | |
| If participants met all three of the fatigue, post-exertional malaise, and unrefreshing sleep or problems sleeping criteria, plus either the cognitive impairment or the orthostatic intolerance criteria, they were sorted into the ME/CFS-like illness category. Otherwise, they were sorted into the No ME/CFS-like illness category. Participants in the ME/CFS-like illness category were further sorted by time of first COVID-19 diagnosis and time of fatigue onset. | | | | |
| First COVID-19 diagnosis | - “Have you ever had COVID-19?   - Yes   - No” - “How many times have you been diagnosed with COVID-19?   - 1   - 2   - 3   - 4   - 5   - 6   - 7   - 8   - 9   - 10 or more **“** | - NAAT-confirmed SARS-CoV-2 - Patient reported positive SARS-CoV-2 antigen test - ICD-10 diagnosis of COVID-19 (J12.82, U07.1, B34.2, B97.2, B97.21, B97.29, J12.81) | To have a first COVID-19 diagnosis, it must be documented in either the medical record or self-reported in the survey | |
| Timing of first COVID-19 | - If self-reported one COVID-19 diagnosis:   - “What year was your most recent COVID-19 diagnosis?”   - “What month was your most recent COVID-19 diagnosis?” | - Date of confirmed test or diagnosis | Medical record was given priority for timing of first COVID-19 diagnosis. If no medical record was found but patient self-reported COVID-19 and reported only one COVID-19 episode, survey timing was used using questions regarding most recent COVID-19. If no medical record was found and patient self-reported more than one COVID-19 episode, timing of first COVID-19 could not be ascertained. | |
| Timing of fatigue | - Timing of fatigue   - “In what year did your fatigue begin?”   - “In what month did your fatigue begin?” | - ICD-09 or ICD-10 diagnosis of fatigue (780.79, 799.3, 323.9, 780.71, R53.1, R53.81, R53.83, R53.82, G93.3) | Self-report was given priority for fatigue, then medical record | |
| Participants with ME/CFS-like illness with fatigue beginning after their first COVID-19 diagnosis were sorted into the ME/CFS-like illness after COVID-19 group, and participants with ME/CFS-like illness pre-dating any COVID-19 diagnosis were sorted into the ME/CFS-like illness without prior COVID-19 group. | | | |  |
