# Supplementary material for: Impact of COVID-19 on myalgic encephalomyelitis/chronic fatigue syndrome-like illness prevalence: A cross-sectional survey
Source: PLoS One. 2024 Sep 18;19(9):e0309810. doi: 10.1371/journal.pone.0309810 (PMC11410243; doi:10.1371/journal.pone.0309810)
Supplement: S2 Table — (DOCX) [file pone.0309810.s002.docx]

**S2 Table. Relationship between exposure, outcome, and primary analysis groups.**

ME/CFS= myalgic encephalomyelitis/chronic fatigue syndrome; COVID-19=coronavirus disease 2019

|  | **ME/CFS-like illness** | **No ME/CFS-like illness^a^** | **Total** |
| --- | --- | --- | --- |
| **COVID-19** | a) 394 | b) 5,606 | 6,000 |
| **No COVID-19** | c) 252 | d) 3,573 | 3,825 |
| **Total** | 646 | 9,179 | 9,825 |
| **Analysis Groups:**  ME/CFS-like illness after COVID-19 (n: 77): subset of a  ME/CFS-like illness without prior COVID-19 (n: 569): c + subset of a  No ME/CFS-like illness^a^ (n: 9,179): b + d | | | |

**^a^**Includes all people without ME/CFS-like illness regardless of whether they have had COVID-19
