# Supplementary material for: Impact of COVID-19 on myalgic encephalomyelitis/chronic fatigue syndrome-like illness prevalence: A cross-sectional survey
Source: PLoS One. 2024 Sep 18;19(9):e0309810. doi: 10.1371/journal.pone.0309810 (PMC11410243; doi:10.1371/journal.pone.0309810)
Supplement: S3 Table — (DOCX) [file pone.0309810.s003.docx]

**S3 Table. Design and non-response weights.**

| **Characteristic** | **Sampling frame/ Initially eligible**  **n (%)^a^** | **Sampled persons n (%)^a^** | **Sampled and eligible persons**  **n (%)^a,b^** | **Eligible respondents prior to weighting**  **n (%)^a^** | **Eligible respondents after sampling and non-response weighting**  **n (%)^c^** | **Standardized mean differences (CI)^d^** |
| --- | --- | --- | --- | --- | --- | --- |
| **Total members** | 2,745,374 | 99,308 | 98,505 | 9,835 | 9,825 |  |
| **Sex** |  |  |  |  |  | 0.10 (0.10, 0.11) |
| Female | 1,460,950 (53) | 54,354 (55) | 53,958 (55) | 6,680 (68) | 6,680 (58) |  |
| Male | 1,284,132 (47) | 44,931 (45) | 44,524 (45) | 3,145 (32) | 3,145 (42) |  |
| Other/Unknown | 292 (<0.1) | 23 (<0.1) | 23 (<0.1) | 10 (0.1) | 0 (0.0) |  |
| **Race & ethnicity** |  |  |  |  |  | 0.09 (0.09, 0.09) |
| Asian | 578,527 (21) | 17,996 (18) | 17,901 (18) | 1,409 (14) | 1,409 (22) |  |
| Black | 209,281 (7.6) | 9,101 (9.2) | 9,001 (9.1) | 571 (5.8) | 570 (6.2) |  |
| Latino/Hispanic | 436,448 (16) | 18,879 (19) | 18,704 (19) | 1,301 (13) | 1,298 (15) |  |
| White | 1,340,164 (49) | 47,962 (48) | 47,572 (48) | 6,167 (63) | 6,162 (52) |  |
| Other/Unknown | 180,954 (6.6) | 5,370 (5.4) | 5,327 (5.4) | 387 (3.9) | 386 (5.4) |  |
| **Age, years** |  |  |  |  |  | 0.08 (0.08, 0.08) |
| 18-29 | 478,186 (17) | 12,236 (12) | 11,447 (12) | 916 (9.3) | 913 (16) |  |
| 30-39 | 511,022 (19) | 15,577 (16) | 15,227 (15) | 1,394 (14) | 1,392 (19) |  |
| 40-49 | 468,348 (17) | 18,390 (19) | 18,270 (19) | 1,670 (17) | 1,668 (16) |  |
| 50-59 | 453,781 (17) | 23,340 (24) | 22,759 (23) | 2,136 (22) | 2,133 (16) |  |
| 60-69 | 425,877 (16) | 19,575 (20) | 19,785 (20) | 2,286 (23) | 2,286 (16) |  |
| 70+ | 408,160 (15) | 10,190 (10) | 11,017 (11) | 1,433 (15) | 1,433 (17) |  |
| **Charlson comorbidity index score** |  |  |  |  |  | 0.14 (0.13, 0.14) |
| No visits prior year | 338,180 (12) | 6,051 (6.1) | 7,122 (7.2) | 281 (2.9) | 281 (8.9) |  |
| Score 0 | 1,653,765 (60) | 57,969 (58) | 54,541 (55) | 5,466 (56) | 5,460 (59) |  |
| Score 1 | 369,425 (14) | 16,883 (17) | 17,361 (18) | 1,963 (20) | 1,959 (15) |  |
| Score 2 | 163,896 (6.0) | 7,454 (7.5) | 7,756 (7.9) | 899 (9.1) | 899 (6.7) |  |
| Score 3+ | 220,108 (8.0) | 10,951 (11) | 11,725 (12) | 1,226 (12) | 1,226 (10) |  |
| **Body mass index, kg/m^2^** |  |  |  |  |  | 0.06 (0.06, 0.06) |
| < 25 | 685,271 (25) | 23,478 (24) | 23,100 (23) | 2,673 (27) | 2,673 (24) |  |
| 25-29.9 | 751,562 (27) | 29,387 (30) | 28,597 (29) | 2,875 (29) | 2,875 (29) |  |
| 30-34.9 | 450,463 (16) | 19,066 (19) | 18,488 (19) | 1,784 (18) | 1,784 (19) |  |
| 35-39.9 | 209,324 (7.6) | 9,309 (9.4) | 9,006 (9.1) | 977 (9.9) | 977 (9.1) |  |
| >= 40 | 154,997 (5.6) | 7,317 (7.4) | 7,035 (7.1) | 838 (8.5) | 838 (7.0) |  |
| Missing | 493,757 (18) | 10,751 (11) | 12,279 (12) | 688 (7.0) | 688 (12) |  |

CI = 95% confidence interval

^a^Unweighted n, unweighted percent

^b^803 sampled persons were found to be ineligible during recruitment

^c,^Unweighted n, weighted percent. Sampling weights are the inverse of the probability of sample selection. Non-response weights were calculated by modeling the probability of survey response within each stratum, adjusting for current age, sex, race, Charlson comorbidity index (CCI) score within the last year, and body mass index (BMI) within the last two years, and taking the inverse of the predicted probability of response. Sampling strata 4-7 were trimmed at the 99^th^ percentile. Sampling and non-response weights were multiplied together to determine final survey weights. 10 respondents were missing data on key elements and were excluded from the final analytic sample

^d^Standardized mean differences are between the sampling frame and respondents after sampling and non-response weighting (column 2 & column 6). The standardized mean difference compares the difference in means. The unit is the pooled standard deviation of the two groups being compared. A standardized mean difference of less than 0.1 is generally considered a negligible difference between groups
