# Supplementary material for: Impact of COVID-19 on myalgic encephalomyelitis/chronic fatigue syndrome-like illness prevalence: A cross-sectional survey
Source: PLoS One. 2024 Sep 18;19(9):e0309810. doi: 10.1371/journal.pone.0309810 (PMC11410243; doi:10.1371/journal.pone.0309810)
Supplement: S4 Table — (DOCX) [file pone.0309810.s004.docx]

**S4 Table. Participants with ME/CFS-like illness from each of the sampling strata.**

| **Sampling stratum number** | **ME/CFS-like illness** | **No ME/CFS-like illness** |
| --- | --- | --- |
| One: Diagnosed ME/CFS | 146 (22.6% ) | 940 (10.2%) |
| Two: Diagnosed Post-COVID-19 conditions | 71 (11.0%) | 509 (5.5%) |
| Three: High risk for ME/CFS | 330 (51.1%) | 3,174 (34.6%) |
| Four: COVID-19 prior to Delta variant predominance | 27 (4.2%) | 737 (8.0%) |
| Five: COVID-19 during Delta variant predominance | 25 (3.9%) | 762 (8.3%) |
| Six: COVID-19 during Omicron variant predominance | 18 (2.8%) | 964 (10.5%) |
| Seven: Those not in prior strata | 29 (4.5%) | 2,093 (22.8%) |
| Total | 646 (100%) | 9,179 (100%) |
