# Supplementary material for: Impact of COVID-19 on myalgic encephalomyelitis/chronic fatigue syndrome-like illness prevalence: A cross-sectional survey
Source: PLoS One. 2024 Sep 18;19(9):e0309810. doi: 10.1371/journal.pone.0309810 (PMC11410243; doi:10.1371/journal.pone.0309810)
Supplement: S5 Table — (DOCX) [file pone.0309810.s005.docx]

**S5 Table. Characteristics of persons with at least one episode of coronavirus disease 2019 (COVID-19), stratified by myalgic encephalomyelitis/chronic fatigue syndrome (ME/CFS)-like illness and COVID-19.**

| **Characteristic** | **All** | | **ME/CFS-like illness after COVID-19** | | **ME/CFS-like illness without prior COVID-19** | | **No ME/CFS-like illness** | |
| --- | --- | --- | --- | --- | --- | --- | --- | --- |
|  | **n^a^** | **% (CI)^a^** | **n^a^** | **% (CI)^a^** | **n^a^** | **% (CI) ^a^** | **n^a^** | **% (CI) ^a^** |
| **Total** | 6,000 |  | 77 |  | 317 |  | 5,606 |  |
| **Source for COVID-19 history** |  |  |  |  |  |  |  |  |
| Electronic health record and self-report | 4,555 | 50 (48, 53) | 60 | 29 (11, 58) | 228 | 59 (38, 77) | 4,267 | 50 (48, 53) |
| Electronic health record only | 91 | 1.3 (0.88, 1.9) | 1 | 0.1 (0.01, 1.0) | 6 | 7.3 (1.2, 33) | 84 | 1.2 (0.82, 1.8) |
| Self-report only | 1,354 | 48 (46, 51) | 16 | 71 (42, 89) | 83 | 34 (17, 56) | 1,255 | 48 (46, 51) |
| **Hospitalization associated with COVID-19** | 297 | 0.6 (0.43, 0.81) | 18 | 3.2 (1.2, 8.1) | 13 | 1.0 (0.38, 2.5) | 266 | 0.6 (0.42, 0.80) |
| **Intensive care unit admission associated with COVID-19** | 70 | 0.2 (0.07, 0.38) | 5 | 1.0 (0.28, 3.2) | 3 | 0.5 (0.09, 2.4) | 62 | 0.2 (0.07, 0.39) |
| **Months since first COVID-19 episode(mean)** |  | 8 (7.9, 8.6) |  | 22 (21, 24) |  | 10 (8.0, 12) |  | 8 (7.8, 8.5) |
| **Predominant variant at time of first COVID-19 episode^b^** |  |  |  |  |  |  |  |  |
| Pre-Delta | 1,380 | 15 (13, 16) | 41 | 74 (34, 94) | 76 | 17 (10, 28) | 1,263 | 14 (13, 16) |
| Delta | 1,383 | 11 (9.5, 12) | 24 | 5.4 (1.9, 15) | 56 | 25 (11, 47) | 1,303 | 11 (9.3, 12) |
| Omicron | 2,996 | 68 (65, 70) | 8 | 1.2 (0.39, 3.5) | 162 | 57 (38, 74) | 2,826 | 68 (66, 70) |
| Unknown | 241 | 6.8 (5.4, 8.4) | 4 | 19 (2.7, 66) | 23 | 0.8 (0.47, 1.5) | 214 | 6.8 (5.4, 8.5) |
| **COVID-19 vaccination status at first COVID-19 episode** |  |  |  |  |  |  |  |  |
| None | 1,916 | 21 (19, 23) | 53 | 96 (87, 99) | 117 | 22 (14, 34) | 1,746 | 21 (19, 22) |
| Primary series only | 1,421 | 18 (16, 20) | 18 | 3.7 (1.1, 11) | 69 | 40 (22, 60) | 1,334 | 18 (16, 20) |
| Primary and at least one additional dose | 2,323 | 53 (51, 56) | 4 | 0.5 (0.14, 1.8) | 106 | 36 (19, 58) | 2,213 | 54 (51, 57) |
| Other | 340 | 7.7 (6.3, 9.4) | 2 | 0.3 (0.05, 1.3) | 25 | 2.4 (0.90, 6.2) | 313 | 7.8 (6.4, 9.6) |

CI = 95% confidence interval

^a^The estimates presented depend on the variable type. Categorical variables present the unweighted n and weighted percent (CI). Continuous variables present the weighted mean (CI)

^b^Predominant variant time periods were defined for analysis purposes as: Pre-Delta, prior to 06/01/2021; Delta, 06/01/2021 – 12/31/2021; Omicron, 01/01/2022 and later
