# Supplementary material for: Impact of COVID-19 on myalgic encephalomyelitis/chronic fatigue syndrome-like illness prevalence: A cross-sectional survey
Source: PLoS One. 2024 Sep 18;19(9):e0309810. doi: 10.1371/journal.pone.0309810 (PMC11410243; doi:10.1371/journal.pone.0309810)
Supplement: S6 Table — (DOCX) [file pone.0309810.s006.docx]

**S6 Table. Reported symptoms in the last 4 weeks among persons with myalgic encephalomyelitis/chronic fatigue syndrome (ME/CFS)-like illness after coronavirus disease 2019 (COVID-19), persons with ME/CFS-like illness without prior COVID-19 and persons without ME/CFS-like illness.**

|  | **Total** | | **ME/CFS-like illness after COVID-19** | | **ME/CFS-like illness without prior COVID-19** | | **No ME/CFS-like illness** | |
| --- | --- | --- | --- | --- | --- | --- | --- | --- |
| **Symptom** | **n^a^** | **% (CI)^a^** | **n^a^** | **% (CI)^a^** | **n^a^** | **% (CI)^a^** | **n^a^** | **% (CI)^a^** |
| **Totals** | 9,825 |  | 77 |  | 569 |  | 9,179 |  |
| Fatigue, tiredness, or exhaustion | 7,693 | 65 (63, 67) | 77 | 100 (100, 100) | 569 | 100 (100, 100) | 7,047 | 64 (62, 66) |
| Unrefreshing sleep or problems sleeping | 7,361 | 64 (62, 66) | 77 | 100 (100, 100) | 569 | 100 (100, 100) | 6,715 | 64 (62, 65) |
| Muscle aches or pains | 6,237 | 52 (50, 54) | 69 | 51 (17, 84) | 513 | 84 (70, 92) | 5,655 | 51 (49, 53) |
| Headaches | 5,174 | 41 (39, 43) | 62 | 66 (23, 93) | 465 | 83 (69, 92) | 4,647 | 40 (38, 42) |
| Joint pain | 5,279 | 41 (39, 43) | 63 | 91 (72, 98) | 462 | 60 (44, 74) | 4,754 | 40 (38, 42) |
| Sinus or nasal congestion | 4,012 | 35 (33, 37) | 41 | 46 (14, 81) | 311 | 45 (31, 61) | 3,660 | 35 (33, 37) |
| Bloating | 3,406 | 26 (24, 28) | 49 | 71 (30, 94) | 343 | 69 (53, 81) | 3,014 | 25 (24, 27) |
| Diarrhea | 2,856 | 22 (21, 24) | 41 | 21 (7.4, 47) | 293 | 40 (27, 55) | 2,522 | 22 (21, 24) |
| Weight gain | 2,940 | 23 (21, 25) | 43 | 69 (29, 92) | 302 | 63 (47, 76) | 2,595 | 22 (21, 24) |
| Post-exertional malaise | 3,766 | 21 (19, 22) | 77 | 100 (100, 100) | 569 | 100 (100, 100) | 3,120 | 19 (18, 21) |
| Muscle weakness | 3,298 | 19 (18, 21) | 55 | 20 (7.1, 45) | 429 | 61 (45, 74) | 2,814 | 19 (17, 20) |
| Stomach or abdominal pain | 2,946 | 20 (18, 21) | 49 | 19 (7.1, 43) | 370 | 63 (47, 76) | 2,527 | 19 (18, 21) |
| Night sweats | 2,618 | 19 (17, 20) | 32 | 42 (12, 80) | 295 | 41 (28, 56) | 2,291 | 18 (17, 20) |
| Constipation | 2,772 | 18 (17, 20) | 36 | 34 (9.9, 71) | 259 | 44 (30, 59) | 2,477 | 18 (16, 19) |
| Forgetfulness or difficulty thinking | 2,852 | 18 (17, 20) | 76 | 100 (97, 100) | 552 | 90 (73, 97) | 2,224 | 17 (16, 19) |
| Numbness | 2,781 | 17 (16, 19) | 44 | 22 (7.2, 52) | 329 | 56 (41, 70) | 2,408 | 16 (15, 18) |
| Shortness of breath | 2,451 | 16 (15, 17) | 59 | 20 (7.3, 44) | 330 | 54 (39, 68) | 2,062 | 15 (14, 17) |
| Bladder problems | 2,108 | 13 (12, 14) | 30 | 13 (4.0, 34) | 234 | 38 (24, 54) | 1,844 | 13 (12, 14) |
| Sensitivity to bright lights | 2,420 | 14 (13, 16) | 44 | 84 (61, 95) | 347 | 55 (39, 70) | 2,029 | 13 (12, 15) |
| Sore throat | 1,418 | 12 (11, 13) | 23 | 12 (3.7, 31) | 153 | 20 (11, 33) | 1,242 | 12 (11, 13) |
| Nausea | 2,012 | 13 (12, 14) | 38 | 13 (4.8, 32) | 309 | 54 (39, 68) | 1,665 | 12 (11, 14) |
| Irregular heartbeat or palpitations | 1,954 | 13 (11, 14) | 44 | 13 (4.7, 30) | 238 | 48 (33, 63) | 1,672 | 12 (11, 13) |
| Sensitivity to noise | 2,317 | 13 (12, 14) | 42 | 62 (26, 88) | 355 | 49 (34, 64) | 1,920 | 12 (11, 14) |
| Loss of appetite | 1,659 | 11 (9.6, 12) | 25 | 13 (4.1, 34) | 270 | 49 (34, 63) | 1,364 | 10 (9.1, 11) |
| Orthostatic intolerance | 1,908 | 10 (9.2, 12) | 43 | 26 (6.0, 66) | 325 | 54 (39, 68) | 1,540 | 9.7 (8.6, 11) |
| Weight loss | 1,135 | 9.1 (8.1, 10) | 11 | 5.4 (1.0, 24) | 103 | 15 (7.3, 28) | 1,021 | 9.1 (8.0, 10) |
| Chest pain | 1,457 | 8.9 (7.9, 10) | 31 | 8.3 (2.8, 22) | 244 | 46 (31, 61) | 1,182 | 8.4 (7.4, 9.5) |
| Sensitivity to smells, foods, medications, or chemicals | 1,844 | 8.3 (7.4, 9.4) | 32 | 34 (8.9, 72) | 304 | 35 (23, 49) | 1,508 | 7.9 (7.0, 8.9) |
| Tender lymph nodes or swollen glands | 954 | 5.9 (5.0, 6.9) | 22 | 14 (4.5, 36) | 153 | 18 (9.6, 30) | 779 | 5.7 (4.8, 6.7) |
| Fever | 580 | 4.3 (3.6, 5.2) | 10 | 6.0 (1.5, 21) | 61 | 11 (4.2, 26) | 509 | 4.2 (3.5, 5.1) |

CI=95% confidence interval

^a^Unweighted n, weighted percent (CI) of respondents who reported experiencing the symptom
