# Supplementary material for: Impact of COVID-19 on myalgic encephalomyelitis/chronic fatigue syndrome-like illness prevalence: A cross-sectional survey
Source: PLoS One. 2024 Sep 18;19(9):e0309810. doi: 10.1371/journal.pone.0309810 (PMC11410243; doi:10.1371/journal.pone.0309810)
Supplement: S7 Table — (DOCX) [file pone.0309810.s007.docx]

**S7 Table. Reported frequent, severe symptoms in the last 4 weeks among persons with myalgic encephalomyelitis/chronic fatigue syndrome (ME/CFS)-like illness after coronavirus disease 2019 (COVID-19), persons with ME/CFS-like illness without prior COVID-19 and persons without ME/CFS-like illness.**

|  | **Total** | | **ME/CFS-like illness after COVID-19** | | **ME/CFS-like illness without prior COVID-19** | | **No ME/CFS-like illness** | |
| --- | --- | --- | --- | --- | --- | --- | --- | --- |
| **Symptom** | **n^a^** | **% (CI)^a^** | **n^a^** | **% (CI) ^a^** | **n^a^** | **% (CI) ^a^** | **n^a^** | **% (CI) ^a^** |
| **Totals** | 9,825 |  | 77 |  | 569 |  | 9,179 |  |
| Unrefreshing sleep or problems sleeping | 4,524 | 31 (29, 33) | 77 | 100 (100, 100) | 569 | 100 (100, 100) | 3,878 | 30 (28, 32) |
| Fatigue, tiredness, or exhaustion | 4,274 | 25 (23, 27) | 77 | 100 (100, 100) | 569 | 100 (100, 100) | 3,628 | 24 (22, 26) |
| Muscle aches or pains | 3,216 | 18 (16, 19) | 51 | 26 (9.3, 54) | 432 | 65 (49, 77) | 2,733 | 17 (15, 18) |
| Joint pain | 2,767 | 16 (15, 17) | 42 | 36 (10, 74) | 367 | 51 (36, 65) | 2,358 | 15 (14, 17) |
| Sinus or nasal congestion | 1,356 | 8.1 (7.1, 9.2) | 17 | 11 (3.4, 31) | 157 | 20 (10, 35) | 1,182 | 7.9 (6.9, 9.1) |
| Muscle weakness | 1,709 | 8.2 (7.2, 9.3) | 37 | 14 (4.8, 35) | 323 | 47 (33, 62) | 1,349 | 7.6 (6.6, 8.7) |
| Weight gain | 1,247 | 7.8 (6.8, 8.9) | 26 | 16 (5.7, 39) | 193 | 39 (26, 55) | 1,028 | 7.3 (6.3, 8.4) |
| Bloating | 1,291 | 7.7 (6.8, 8.8) | 33 | 17 (6.1, 40) | 212 | 45 (31, 61) | 1,046 | 7.2 (6.2, 8.2) |
| Headaches | 1,505 | 7.9 (6.9, 9.0) | 40 | 38 (11, 74) | 275 | 64 (49, 77) | 1,190 | 7 (6.1, 8.0) |
| Forgetfulness or difficulty thinking | 1,649 | 8.2 (7.2, 9.3) | 74 | 98 (89, 100) | 538 | 89 (73, 96) | 1,037 | 6.8 (5.9, 7.9) |
| Post-exertional malaise | 2,005 | 7.7 (6.7, 8.8) | 77 | 100 (100, 100) | 569 | 100 (100, 100) | 1,359 | 6.1 (5.3, 7.1) |
| Numbness | 1,171 | 5.4 (4.6, 6.3) | 23 | 12 (3.8, 33) | 193 | 40 (26, 56) | 955 | 4.9 (4.1, 5.8) |
| Bladder problems | 938 | 4.9 (4.2, 5.8) | 24 | 12 (3.6, 33) | 137 | 29 (17, 45) | 777 | 4.6 (3.9, 5.4) |
| Stomach or abdominal pain | 1,035 | 5.0 (4.2, 5.9) | 25 | 12 (3.7, 31) | 208 | 39 (25, 54) | 802 | 4.5 (3.8, 5.3) |
| Sensitivity to bright lights | 1,054 | 4.8 (4.1, 5.7) | 27 | 24 (5.1, 66) | 222 | 28 (18, 43) | 805 | 4.4 (3.7, 5.3) |
| Constipation | 948 | 4.6 (3.9, 5.4) | 16 | 6.7 (1.9, 21) | 139 | 23 (12, 40) | 793 | 4.3 (3.6, 5.1) |
| Night sweats | 888 | 4.6 (3.9, 5.5) | 17 | 5.4 (1.3, 20) | 163 | 28 (16, 44) | 708 | 4.3 (3.6, 5.1) |
| Sensitivity to noise | 1,044 | 4.3 (3.6, 5.1) | 24 | 3.1 (1.3, 7.6) | 235 | 29 (18, 42) | 785 | 3.9 (3.3, 4.7) |
| Diarrhea | 724 | 3.7 (3.0, 4.5) | 16 | 5.3 (1.7, 16) | 131 | 26 (16, 41) | 577 | 3.3 (2.7, 4.1) |
| Loss of appetite | 594 | 3.6 (2.9, 4.4) | 8 | 1.3 (0.44, 3.8) | 152 | 36 (23, 51) | 434 | 3.1 (2.5, 3.9) |
| Shortness of breath | 721 | 3.2 (2.6, 4.0) | 35 | 6.1 (2.5, 14) | 162 | 30 (18, 46) | 524 | 2.9 (2.3, 3.6) |
| Sensitivity to smells, foods, medications, or chemicals | 895 | 2.9 (2.4, 3.5) | 22 | 7.3 (2.2, 22) | 217 | 23 (14, 36) | 656 | 2.6 (2.1, 3.2) |
| Nausea | 487 | 2.4 (1.9, 3.0) | 17 | 4.1 (1.3, 12) | 128 | 35 (22, 52) | 342 | 1.9 (1.4, 2.5) |
| Irregular heartbeat or palpitations | 361 | 1.8 (1.3, 2.3) | 20 | 6.2 (1.9, 19) | 79 | 27 (15, 44) | 262 | 1.4 (1.0, 1.9) |
| Orthostatic intolerance | 406 | 1.8 (1.3, 2.4) | 19 | 4.4 (1.4, 13) | 160 | 34 (21, 50) | 227 | 1.3 (0.92, 1.9) |
| Chest pain | 259 | 1.4 (1.0, 2.0) | 10 | 1.5 (0.52, 4.2) | 76 | 17 (8.0, 32) | 173 | 1.2 (0.83, 1.7) |
| Weight loss | 206 | 1.3 (0.91, 1.8) | 3 | 4.3 (0.60, 25) | 36 | 7.8 (2.7, 20) | 167 | 1.2 (0.82, 1.7) |
| Sore throat | 191 | 0.9 (0.61, 1.3) | 6 | 1.2 (0.39, 3.9) | 42 | 3.2 (1.0, 9.7) | 143 | 0.9 (0.58, 1.3) |
| Tender lymph nodes or swollen glands | 212 | 0.9 (0.61, 1.4) | 6 | 3.9 (0.66, 20) | 69 | 8.7 (4.0, 18) | 137 | 0.8 (0.50, 1.3) |
| Fever | 51 | 0.3 (0.16, 0.71) | 1 | <0.1 (0.01, 0.74) | 7 | 5.2 (0.89, 25) | 43 | 0.3 (0.12, 0.60) |

CI=95% confidence interval

^a^Unweighted n, weighted percent (CI) of respondents who reported experiencing the symptom “a good bit of the time” to “all of the time” and at a “moderate” to “very severe” intensity
