# Supplementary material for: Impact of COVID-19 on myalgic encephalomyelitis/chronic fatigue syndrome-like illness prevalence: A cross-sectional survey
Source: PLoS One. 2024 Sep 18;19(9):e0309810. doi: 10.1371/journal.pone.0309810 (PMC11410243; doi:10.1371/journal.pone.0309810)
Supplement: S1 Appendix — (DOCX) [file pone.0309810.s008.docx]

**S1 Appendix. Survey.**

**Well Being Study**

**Thank you for taking part in the Well Being Study. As part of this study, you will answer questions about your emotional, mental and physical health. This survey is the first of two online surveys. Please complete this first one now. We will remind you in one year by email, text, or phone when it is time for your next survey. Answering these questions will take approximately 30 minutes. You can leave and come back by clicking “Save and Return Later”. Your answers will be saved. You will receive a $10 Amazon e-gift card after submitting each survey.**

**Please click Next Page to go to the survey.**

**If you have any questions, you may contact us at WellBeingStudy@kp.org or 510-891-3668.**

**Thank you,**

**Your Well Being Study Team**

# Your Health And Quality Of Life

**The following questions are about your health and quality of life.**

1. **In general, would you say your health is:**
2. Excellent
3. Very Good
4. Good
5. Fair
6. Poor
7. **Compared to one year ago, how would you rate your health in general now?**
8. Much better than one year ago
9. Somewhat better than one year ago
10. About the same as one year ago
11. Somewhat worse than one year ago
12. Much worse than one year ago

|  | **The following items are about activities you might do during a typical day. Does your health now limit you in these activities? If so, how much?** | **Please mark the appropriate box.** | | |
| --- | --- | --- | --- | --- |
|  |  | **Yes, limited a lot** | **Yes, limited a little** | **No, not limited at all** |
| 3. | **Vigorous activities, such as running, lifting heavy objects, participating in strenuous sport** |  |  |  |
| 4. | **Moderate Activities, such as moving a table, pushing a vacuum cleaner, bowling, or playing golf** |  |  |  |
| 5. | **Lifting or carrying groceries** |  |  |  |
| 6. | **Climbing several flights of stairs** |  |  |  |
| 7. | **Climbing one flight of stairs** |  |  |  |
| 8. | **Bending, kneeling, or stooping** |  |  |  |
| 9. | **Walking more than a mile** |  |  |  |
| 10. | **Walking several hundred yards** |  |  |  |
| 11. | **Walking one hundred yards** |  |  |  |
| 12. | **Bathing or dressing yourself** |  |  |  |

|  | **During the past 4 weeks, have you had any of the following problems with your work or other regular daily activities as a result of your physical health?** | **Please mark the appropriate box.** | | | | |
| --- | --- | --- | --- | --- | --- | --- |
|  |  | **All of the time** | **Most of the time** | **Some of the time** | **A little of the time** | **None of the time** |
| 13. | **Cut down on the amount of time you spent on work or other activities** |  |  |  |  |  |
| 14. | **Accomplished less than you would like** |  |  |  |  |  |
| 15. | **Were limited in the kind of work or other activities** |  |  |  |  |  |
| 16. | **Had difficulty performing the work or other activities (for example, it took extra effort)** |  |  |  |  |  |

|  | **During the past 4 weeks, have you had any of the following problems with your work or other regular daily activities as a result of any emotional problems (such as feeling depressed or anxious)?** | **Please mark the appropriate box.** | | | | |
| --- | --- | --- | --- | --- | --- | --- |
|  |  | **All of the time** | **Most of the time** | **Some of the time** | **A little of the time** | **None of the time** |
| 17. | **Cut down on the amount of time you spent on work or other activities** |  |  |  |  |  |
| 18. | **Accomplished less than you would like** |  |  |  |  |  |
| 19. | **Did your work or activities less carefully than usual** |  |  |  |  |  |

1. **During the past 4 weeks, to what extent has your physical health or emotional problems interfered with your normal social activities with family, friends, neighbors, or groups?**
2. Not at all
3. Slightly
4. Moderately
5. Quite a bit
6. Extremely
7. **How much bodily pain have you had during the past 4 weeks?**
8. None
9. Very mild
10. Mild
11. Moderate
12. Severe
13. Very severe
14. **During the past 4 weeks, how much did pain interfere with your normal work (including both work outside the home and housework)?**
15. None
16. A little bit
17. Moderately
18. Quite a bit
19. Extremely

**These questions are about how you feel and how things have been with you during the past 4 weeks. For each question, please give the one answer that comes closest to the way you have been feeling.**

|  | **How much of the time during the past 4 weeks…** | **Please mark the appropriate box.** | | | | |
| --- | --- | --- | --- | --- | --- | --- |
|  |  | **All of the time** | **Most of the time** | **Some of the time** | **A little of the time** | **None of the time** |
| **23.** | **Did you feel full of life?** |  |  |  |  |  |
| **24.** | **Have you been very nervous?** |  |  |  |  |  |
| **25.** | **Have you felt so down in the dumps that nothing could cheer you up?** |  |  |  |  |  |
| **26.** | **Have you felt calm and peaceful?** |  |  |  |  |  |
| **27.** | **Did you have a lot of energy?** |  |  |  |  |  |
| **28.** | **Have you felt downhearted and depressed?** |  |  |  |  |  |
| **29.** | **Did you feel worn out?** |  |  |  |  |  |
| **30.** | **Have you been happy?** |  |  |  |  |  |
| **31.** | **Did you feel tired?** |  |  |  |  |  |

**32. During the past 4 weeks, how much of the time has your physical health or emotional problems interfered with your social activities (like as visiting friends, relatives, etc.)?**

- 1. All of the time
  2. Most of the time
  3. Some of the time
  4. A little of the time
  5. None of the time

**How TRUE OR FALSE is each of the following statements for you?**

|  | **How TRUE OR FALSE is each of the following statements for you?** | **Please mark the appropriate box.** | | | | |
| --- | --- | --- | --- | --- | --- | --- |
|  |  | **Definitely true** | **Mostly true** | **Don’t know** | **Mostly false** | **Definitely false** |
| **33.** | **I seem to get sick a little easier than other people.** |  |  |  |  |  |
| **34.** | **I am as healthy as anybody I know.** |  |  |  |  |  |
| **35.** | **I expect my health to get worse.** |  |  |  |  |  |
| **36.** | **My health is excellent.** |  |  |  |  |  |

# YOUR MOOD AND FEELINGS

**The following questions are about your mood and feelings.**

|  | **Over the last 2 weeks, how often have you been bothered by any of the following problems?** | **Not at all** | **Several days** | **More than half of the days** | **Nearly every day** |
| --- | --- | --- | --- | --- | --- |
| **1.** | **Little interest or pleasure in doing things** | If no problems are marked, question  **11** must be skipped |  | If **one or more questions** are answered with ‘Several days’, ‘More than half of the days’ or ‘Nearly every day’, then **question 11** **must be asked.** |  |
| **2.** | **Feeling down, depressed, or hopeless** |  |  |  |  |
| **3.** | **Trouble falling or staying asleep, or sleeping too much** |  |  |  |  |
| **4.** | **Feeling tired or having little energy** |  |  |  |  |
| **5.** | **Poor appetite or overeating** |  |  |  |  |
| **6.** | **Feeling bad about yourself – or that you are a failure or have let yourself or your family down** |  |  |  |  |
| **7.** | **Trouble concentrating on things, such as reading the newspaper or watching television** |  |  |  |  |
| **8.** | **Moving or speaking so slowly that other people could have noticed. Or the opposite -- being so fidgety or restless that you have been moving around a lot more than usual** |  |  |  |  |
| **9.** | **Feeling nervous, anxious or on edge** |  |  |  |  |
| **10.** | **Not being able to stop or control worrying** |  |  |  |  |

**11. If you checked off any problems, how difficult have these problems made it for you to do your work, take care of things at home, or get along with other people?**

1. Not difficult at all
2. Somewhat difficult
3. Very difficult
4. Extremely difficult

# Your Symptoms

**We will now ask you about any symptoms that you might have experienced in the past 4 weeks. If you have had a symptom in the past 4 weeks, we will ask some follow up questions.**

**SYMPTOM LIBRARY OVERVIEW**

If Question 1 is answered ‘YES’ , then needs to answer questions 2, 3, and 4 for that symptom.

|  |  | **Question 1** | **Question 2** | **Question 3** | **Question 4** |
| --- | --- | --- | --- | --- | --- |
| **Symtpom #** | **Symptom Name** | During the **past 4 weeks**, have you had this symptom?  *Yes/No* | During the **past 4 weeks**, **how often** have you had difficulty with this symptom? **Select one.**  *A little of the time; Some of the time; Aa good bit of the time; Most of the time; All of the time* | During the **past 4 weeks,** **how bad** was this [symptom]?  **Select one.**  *Very mild, mild, moderate, severe, very severe* | **Prior to this past 4 weeks**, for **how long** had you had difficulty with [symptom]? **Select one.**  *Less than 6 months;*  *6 months up to 1 year;*  *1 year up to 3 years;*  *3 years up to 5 years*  *5 years up to 10 years; 10 or more years* |
| 1 | Fever |  |  |  |  |
| 2 | Night sweats |  |  |  |  |
| 3 | Headaches |  |  |  |  |
| 4 | Sinus or nasal congestion |  |  |  |  |
| 5 | Sore throat |  |  |  |  |
| 6 | Tender lymph nodes or swollen glands |  |  |  |  |
| 7 | Muscle aches or pains |  |  |  |  |
| 8 | Muscle weakness |  |  |  |  |
| 9 | Numbness |  |  |  |  |
| 10 | Joint pain |  |  |  |  |
| 11 | Nausea |  |  |  |  |
| 12 | Loss of appetite |  |  |  |  |
| 13 | Stomach or abdominal pain |  |  |  |  |
| 14 | Bloating |  |  |  |  |
| 15 | Diarrhea |  |  |  |  |
| 16 | Constipation |  |  |  |  |
| 17 | Weight loss |  |  |  |  |
| 18 | Weight gain |  |  |  |  |
| 19 | Chest pain |  |  |  |  |
| 20 | Irregular heartbeat or palpitations |  |  |  |  |
| 21 | Shortness of breath |  |  |  |  |
| 22 | Bladder problems |  |  |  |  |
| 23 | Sensitivity to noise |  |  |  |  |
| 24 | Sensitivity to bright lights |  |  |  |  |
| 25 | Sensitivity to smells, foods, medications, or chemicals |  |  |  |  |

**1. During the past 4 weeks, have you had any [symptom 1 from table]?**

- 1. Yes (skip to question 2)
  2. No (skip to next symptom question)

**2. During the past 4 weeks, how often have you had difficulty with [symptom]?**

- 1. A little of the time
  2. Some of the time
  3. A good bit of the time
  4. Most of the time
  5. All of the time

**3. During the past 4 weeks, how bad was your difficulty with [symptom]?**

- 1. Very mild
  2. Mild
  3. Moderate
  4. Severe
  5. Very severe

**4. For how long had you had difficulty with [symptom]?**

1. Less than 6 months
2. 6 months up to 1 year
3. 1 year up to 3 years
4. 3 years up to 5 years
5. 5 years up to 10 years
6. 10 or more years

# Your Energy

**Please tell us if you have had any of the symptoms listed below in the past 4 weeks.**

1. **During the past 4 weeks, have you had fatigue, tiredness, or exhaustion? Select one.**
2. Yes (answer questions 2, 3, 4)
3. No (skip to question 5)
4. **During the past 4 weeks, how often have you had fatigue, tiredness, or exhaustion? Select one.**
5. A little of the time
6. Some of the time
7. A good bit of the time
8. Most of the time
9. All of the time
10. **During the past 4 weeks, how bad was your fatigue, tiredness, or exhaustion? Select one.**
11. Very mild
12. Mild
13. Moderate
14. Severe
15. Very severe
16. **How long have you had fatigue, tiredness, or exhaustion? Select one.**
17. Less than 6 months
18. 6 months up to 1 year
19. 1 year up to 3 years
20. 3 years up to 5 years
21. 5 years up to 10 years
22. 10 or more years
23. **During the past 4 weeks, have you been unusually fatigued or unwell for at least one day after exerting yourself in any way? Select one.**
24. Yes
25. No
26. **During the past 4 weeks, how often have you had unusual fatigue after exertion? Select one.**
27. A little of the time
28. Some of the time
29. A good bit of the time
30. Most of the time
31. All of the time
    - 1. **During the past 4 weeks, how bad was your unusual fatigue after exertion? Select one.**
32. Very mild
33. Mild
34. Moderate
35. Severe
36. Very severe
37. **How long have you had unusual fatigue after exertion? Select one.**
38. Less than 6 months
39. 6 months up to 1 year
40. 1 year up to 3 years
41. 3 years up to 5 years
42. 5 years up to 10 years
43. 10 or more years
44. **During the past 4 weeks, has unrefreshing sleep been a problem for you? Select one.**
45. Yes
46. No
47. **During the past 4 weeks, how often have you had unrefreshing sleep?**
48. A little of the time
49. Some of the time
50. A good bit of the time
51. Most of the time
52. All of the time
53. **During the past 4 weeks, how much of a problem was unrefreshing sleep?**
54. Very mild
55. Mild
56. Moderate
57. Severe
58. Very severe
59. **How long had you had unrefreshing sleep?**
60. Less than 6 months
61. 6 months up to 1 year
62. 1 year up to 3 years
63. 3 years up to 5 years
64. 5 years up to 10 years
65. 10 or more years
66. **During the past 4 weeks, have you had problems getting to sleep, sleeping through the night, or waking up on time? Select one.**
67. Yes
68. No
69. **During the past 4 weeks, how often have you had sleeping problems? Select one.**
70. A little of the time
71. Some of the time
72. A good bit of the time
73. Most of the time
74. All of the time
75. **During the past 4 weeks, how bad were these sleeping problems? Select one.**
76. Very mild
77. Mild
78. Moderate
79. Severe
80. Very severe
81. **How long have you had sleeping problems? Select one.**
82. Less than 6 months
83. 6 months up to 1 year
84. 1 year up to 3 years
85. 3 years up to 5 years
86. 5 years up to 10 years
87. 10 or more years
88. **During the past 4 weeks, have you had forgetfulness or memory problems that caused you to substantially cut back on your activities? Select one.**
89. Yes
90. No
91. **During the past 4 weeks, how often have you had forgetfulness or memory problems? Select one.**
92. A little of the time
93. Some of the time
94. A good bit of the time
95. Most of the time
96. All of the time

**19. During the past 4 weeks, how bad were your** **forgetfulness or memory problems? Select one.**

1. Very mild
2. Mild
3. Moderate
4. Severe
5. Very severe
   - 1. **How long have you had forgetfulness or memory problems? Select one.**
   1. Less than 6 months
   2. 6 months up to 1 year
   3. 1 year up to 3 years
   4. 3 years up to 5 years
6. 5 years up to 10 years
7. 10 or more years
   - 1. **During the past 4 weeks, have you had difficulty with thinking or concentrating that caused you to substantially cut back on your activities? Select one.**
8. Yes
9. No
   - 1. **During the past 4 weeks, how often have you had difficulty with thinking or concentrating? Select one.**
10. A little of the time
11. Some of the time
12. A good bit of the time
13. Most of the time
14. All of the time
    - 1. **During the past 4 weeks, how severe was your difficulty with thinking or concentrating? Select one.**
15. Very mild
16. Mild
17. Moderate
18. Severe
19. Very severe
    - 1. **How long have you had difficulty with thinking or concentrating? Select one.**
    1. Less than 6 months
    2. 6 months up to 1 year
    3. 1 year up to 3 years
    4. 3 years up to 5 years
20. 5 years up to 10 years
21. 10 or more years
    - 1. **During the past 4 weeks, have you had dizziness or fainting problems? Select one.**
    1. Yes
22. No
    - 1. **During the past 4 weeks, how often have you had dizziness or fainting problems? Select one.**
23. A little of the time
24. Some of the time
25. A good bit of the time
26. Most of the time
27. All of the time

**27. During the past 4 weeks, how bad was your dizziness or fainting problems? Select one.**

1. Very mild
2. Mild
3. Moderate
4. Severe
5. Very severe

**28. How long have you had dizziness or fainting problems? Select one.**

- 1. Less than 6 months **(**skip to part 5, or 6, or 7, or 8 based on screener answers)
  2. 6 months up to 1 year (skip to part 5, or 6, or 7, or 8 based on screener answers)
  3. 1 year up to 3 years
  4. 3 years up to 5 years
  5. 5 years up to 10 years
  6. 10 or more years

*SECTION BREAK ONLINE – USE MOVE FORWARD AND BACK BUTTOMS TO NAVIGATE TO NEXT SECTION*

# Your Energy - Continued

**In previous sections you indicated that you may have had fatigue,** **tiredness or exhaustion or felt unusually fatigued at least one day after exerting yourself. The next questions are related to the fatigue, tiredness or exhaustion you described above.**

1. **In what year did your fatigue begin? Select one.**

Dropdown answer choices: 1990, 1991, …..2019, 2020, 2021, 2022, 2023, 2024, and 'I don't know’

1. **In what month did your fatigue begin? Select one.**

Dropdown answer choices: January, February, March, April, May, June, July, August, September, October, November, December, and 'I don't know'.

1. **When the fatigue started, would you say that it came on all of a sudden, or slowly over time? Select one.**
2. All of a sudden
3. Slowly over time
4. Not applicable
5. Don't know
6. **When you experience fatigue, does rest make your fatigue better? Select one.**
7. Yes, a lot
8. Yes, a little
9. No, not very much
10. No, not at all
11. **Has your fatigue substantially limited your ability to pursue your work, educational, social, or recreational activities? Select one.**
12. Yes
13. No
14. Not applicable
15. **Have you ever consulted a medical doctor or health professional about your fatigue? Select one.**
16. Yes
17. No
18. Not applicable
19. **Do you currently have a medical doctor or other health professional overseeing your fatigue? Select one.**
20. Yes
21. No
22. Not applicable
23. **Have you been diagnosed with a medical condition that causes fatigue (or that your health professional told you causes fatigue)? Select one.**
24. Yes
25. No
26. Not applicable
27. **Have you ever been diagnosed with Chronic Fatigue Syndrome or Myalgic Encephalomyelitis? Select one.**
28. Yes
29. No
30. **If yes, what year were you diagnosed Chronic Fatigue Syndrome or Myalgic Encephalomyelitis? Type the year in the text box (for example: 2020).**

**Year _____**

1. **Do you currently have a diagnosis of Chronic Fatigue Syndrome or Myalgic Encephalomyelitis? Select one.**
2. Yes
3. No
4. **Who diagnosed you with Chronic Fatigue Syndrome or Myalgic Encephalomyelitis? Select one.**
5. Medical doctor or other health professional
6. Alternative practitioner
7. Self-diagnosed
8. **How would you describe the course of your fatigue? Select one.**
9. Constantly getting worse
10. Constantly improving
11. Persisting (no change)
12. Relapsing & remitting (having “good” periods with no symptoms & “bad” periods)
13. Fluctuating (symptoms periodically get better and get worse, but never disappear completely)
14. No Symptoms/I am not ill
15. **What do you think is the cause of your problem with fatigue? Select one.**
16. Definitely physical
17. Mainly physical
18. Equally physical and psychological
19. Mainly psychological
20. Definitely psychological

# Your Orthostatic Symptoms

**In a previous section you indicated that you had dizziness or fainting problems in the past 4 weeks. We will now ask you some follow-up questions about these symptoms.**

1. **How often do you experience dizziness or fainting symptoms?**
2. Never or rarely
3. Sometimes (Two or three times)
4. Often (About once a day)
5. Always
6. **How severe are your dizziness or fainting symptoms when you stand up?**
7. I do not experience dizziness or fainting symptoms
8. Mild dizziness or fainting symptoms
9. Moderate dizziness or fainting symptoms (sometimes have to sit back down for relief)
10. Severe dizziness or fainting symptoms (frequently sit back down for relief)
11. Very sever dizziness or fainting symptoms (regularly faint if not sitting back down)
12. **How often have you experienced dizziness or fainting symptoms under certain conditions, such as prolonged standing, a meal, exertion (e.g., walking), or when exposed to heat (e.g., hot day, hot bath, hot shower)?**
13. I never or rarely experience dizziness or fainting symptoms
14. I sometimes experience dizziness or fainting symptoms
15. I often experience dizziness or fainting symptoms
16. I usually experience dizziness or fainting symptoms
17. I always experience dizziness or fainting symptoms
18. **How do your dizziness or fainting symptoms interfere with activities of daily living, e.g., work, chores, dressing, bathing, etc.).**
19. Do not interfere with activities of daily living
20. Mildly interfere with activities of daily living
21. Moderately interfere with activities of daily living
22. Severely interfere with activities of daily living
23. Extremely interfere (I am bed or wheelchair bound)
24. **On most occasions, how long can you stand before experiencing dizziness or fainting symptoms?**
25. I can stand as long as necessary
26. I can stand more than 15 minutes
27. I can stand 5-14 minutes
28. I can stand 1-4 minutes
29. I can stand less than 1 minute

*SECTION BREAK ONLINE – USE MOVE FORWARD AND BACK BUTTOMS TO NAVIGATE TO NEXT SECTION*

# Your Cognitive Function

**We will now ask you some questions about your cognitive function.**

**In the past 7 days, how often have you had difficulty with your memory or thinking?**

|  | **In the past 7 days….** | **Never** | **Rarely (Once)** | **Sometimes (Two or three times)** | **Often (About once a day)** | **Very Often (Several times a day)** |
| --- | --- | --- | --- | --- | --- | --- |
| 1. | **My thinking has been slow…** |  |  |  |  |  |
| 2. | **It has seemed like my brain was not working as well as usual…** |  |  |  |  |  |
| 3. | **I have had to work harder than usual to keep track of what I was doing…** |  |  |  |  |  |
| 4. | **I have had trouble shifting back and forth between different activities that require thinking…** |  |  |  |  |  |

*SECTION BREAK ONLINE – USE MOVE FORWARD AND BACK BUTTOMS TO NAVIGATE TO NEXT SECTION*

# Your Covid History

**This next section is about COVID-19. We will ask if you have ever been diagnosed with COVID-19. If you have been diagnosed with COVID-19, we will ask some follow-up questions.**

1. **Have you ever had COVID-19? Select one.**
   1. Yes
2. No
3. **How many times have you been diagnosed with COVID-19?**
   1. 1
   2. 2
   3. 3
   4. 4
   5. 5
   6. 6
   7. 7
   8. 8
   9. 9
   10. 10 or more
4. **What year was your most recent COVID-19 diagnosis? Select one.**

Dropdown answer choices: 2020, 2021, 2022, 2023, 2024, and 'I don't know’

1. **What month was your most recent COVID-19 diagnosis? Select one.**

Dropdown answer choices: January, February, March, April, May, June, July, August, September, October, November and 'I don't know’

**5. How were you diagnosed with COVID-19 for your most recent infection? Select one.**

1. I had a self-administered positive nose or throat swab test for COVID-19 at home
2. I had a positive nose or throat swab test for COVID-19 *at a clinical facility, such as a lab, doctor’s office or pharmacy*

**6. Did you receive any medical care for your COVID-19? Select one.**

1. Yes
2. No

**7. What kind of medical care did you receive for your COVID-19? Mark all that apply.**

1. None
2. Telephone visit with a doctor or medical provider
3. Video visit with a doctor or medical provider
4. Office visit with a doctor or medical provider
5. Emergency room visit
6. Hospital admission
7. Admission to an intensive care unit (ICU)
8. Other: Please specify_____

**8. Did you have any symptoms when you were recently had COVID-19? Select one.**

1. Yes
2. No

***9*. What symptoms did you have when you *most recently had COVID-19?* Mark all that apply.**

o None

o Fever

o Chills

o Cough

o Shortness of breath

o Chest Pain

o Sore throat

o Headache

o Muscle or body aches

o Runny nose

o Fatigue

o Confusion

o Diarrhea

o Nausea or vomiting

o Loss of taste

o Loss of smell

o Other specify

***10.*  How would you describe your symptoms now?**

1. I feel better and am completely recovered
2. I feel better but have not completely recovered
3. I feel the same as when I had COVID-19
4. I feel worse now than when I had COVID-19

**11. What symptoms are you currently experiencing that you believe are related to most recent COVID-19 infection? Mark all that apply.**

|  |  |
| --- | --- |
| **Symptoms** | **Related to COVID-19 infection? Yes/No** |
| None |  |
| Fever |  |
| Chills |  |
| Fatigue, tiredness or exhaustion |  |
| Unusual fatigue after exerting yourself |  |
| Night sweats |  |
| Headaches |  |
| Sinus or nasal Symptoms |  |
| Sore throat |  |
| Cough |  |
| Tender lymph nodes or swollen glands |  |
| Muscle aches and pains |  |
| Muscle weakness |  |
| Numbness |  |
| Joint pain |  |
| Nausea |  |
| No appetite |  |
| Stomach or abdominal pain |  |
| Bloating |  |
| Diarrhea |  |
| Constipation |  |
| Weight Loss |  |
| Weight gain |  |
| Chest pain |  |
| Irregular heartbeat or palpitations |  |
| Shortness of breath |  |
| Bladder problems |  |
| Sensitivity to noise |  |
| Sensitivity to bright lights |  |
| Sensitivity to smells, foods, medications, or chemicals |  |
| Unrefreshing sleep |  |
| Problems getting to sleep, sleeping through the night, or waking up on time |  |
| Forgetfulness or memory problems |  |
| Other - Specify |  |

*SECTION BREAK ONLINE – USE MOVE FORWARD AND BACK BUTTOMS TO NAVIGATE TO NEXT SECTION*

# DEMOGRAPHICS

**This section asks some general questions about yourself.**

1. **What is your gender? Select one.**
2. Female
3. Male
4. Transgender Female
5. Transgender Male
6. Gender Variant/Non-Conforming
7. Not listed _________________
8. Prefer not to answer
9. **Which of the following best represents how you think of yourself? Select one.**
10. Gay or homosexual
11. Bisexual or pansexual
12. Straight or heterosexual
13. Something else
14. Prefer not to answer
15. I don’t know
16. **What describes your race and ethnicity? Select all that apply.**

White or of European descent

Middle Eastern/North African Arab

African American

Other Black, please specify: ___________________

Mexican or Central American ancestry

Other Hispanic/Latino, please specify: _________________

Filipino

Chinese or Taiwanese

Korean

Japanese

South Asian (Indian, Pakistani, Afghani, etc.)

Southeast Asian, please specify: __________________

Iranian or Persian

Other Asian, please specify: ______________________

Native Hawaiian or Pacific Islander

Native American Indian or Alaska Native

Other, please specify: ___________________________

1. **What is the highest level of school you completed? Select one.**
2. 8th grade or less (primary or middle school)
3. 9th - 11th grade (some high school)
4. 12th grade (high school graduate or G.E.D.)
5. Technical/trade school certificate
6. Some college (no degree)
7. Associate’s Degree (e.g., AA, AS)
8. Bachelor’s Degree (e.g., BA), teaching credential
9. Graduate or professional degree (e.g., MA, MD)
10. **What is your current work status? Select all that apply.**

Working for pay → How many hours/week? ___

Self-employed → How many hours/week? ___

Unemployed or laid off

Unable to work due to health/disability

Retired

Homemaker, parent, or unpaid caregiver

Part-time or full-time student

Do volunteer work at least once a week

1. **Which of the following best describes your current relationship status? Select one.**
   1. Married
   2. Widowed
   3. Divorced
   4. Separated
   5. In a domestic partnership or civil union
   6. Single, but cohabiting with significant other
   7. Single, living alone
2. **How many total people – adults and children – currently live in your household, including yourself?**

Please enter a number: ____________

1. **Which of the following best describes your total annual household (family) income from**

**all sources in the past year before taxes?**

1. Less or equal to $25,000 per year
2. $25,001 - $35,000 per year
3. $35,001 - $50,000 per year
4. $50,001 - $75,000 per year
5. $75,001 - $100,000 per year
6. $100,001 - $150,000 per year
7. $150,001 - $200,000 per year
8. More than $200,000 per year
9. Don’t know
10. Prefer not to answer

**Thank you for taking the Well Being Survey. You will receive your Amazon e- gift card code by email in 2-3 business days.**

**Sincerely, Your Well Being Study Team**
