# Supplementary material for: Impact of COVID-19 on myalgic encephalomyelitis/chronic fatigue syndrome-like illness prevalence: A cross-sectional survey
Source: PLoS One. 2024 Sep 18;19(9):e0309810. doi: 10.1371/journal.pone.0309810 (PMC11410243; doi:10.1371/journal.pone.0309810)
Supplement: S2 Appendix — (DOCX) [file pone.0309810.s009.docx]

**S2 Appendix. Predictive model.**

Seventy-one covariates were initially used to create the predictive model including age, sex, race/ethnicity, presence of a general fatigue diagnosis in the last year, presence of a fatigue diagnosis prior to one year ago, 30 comorbidities from the Elixhauser Comorbidity Index, and ICD-09 and ICD-10 codes for health conditions including the following: adjustment disorders, agoraphobia, alcohol related disorders, allergies, anxiety disorders, chronic interstitial cystitis, chronic pain, chronic tenson-type headache, cognitive impairment, depressive disorders, drug related disorders, eating disorders, Ehlers-Danlos syndrome, endometriosis, headache, irritable bowel sydnrome, low back pain, malingerer consicous simulation, migraine, myositis myalgia and fibromyalgia, nonrheumatic mitral valve prolapse, obsessive-compulsive disorder, orthostatic intolerance, other systemic involvement of connective tissue, pelvic and peritoneal pain, personality disorders, post-traumatic stress disorder, Raynaud’s syndrome, reactions to severe stress, Sicca syndrome, sleep disorders, somatoform disorders, symptoms and signs involving general sensations and perceptions, temporomandibular joint disorders, unspecified mood affective disorder, and vulvodynia (full ICD code list available upon request). The outcome of the model was ME/CFS diagnosis in the medical record used as a proxy for ME/CFS.

The model was trained using LASSO regression. Ten-fold cross validation was used to select the optimal penalty term lambda (0.0000719). After applying the penalty, the fitted model contained non-zero coefficients for the following covariates: age, race/ethnicity, presence of a general fatigue diagnosis in the last year, presence of a fatigue diagnosis prior to one year ago, Elixhauser anemia, Elixhauser chronic pulmonary disease, Elixhauser drug abuse, Elixhauser fluid electrolyte disorders, Elixhauser hypertension, Elixhauser hypothyroidism, Elixhauser liver disease, Elixhauser obesity, Elixhauser renal failure, Elixhauser rheumatoid arthritis collagen, Elixhauser solid tumor without metastasis, Elixhauser weight loss, Elixhauser cardiac arrythmia, Elixhauser other neurological disorders, Elixhauser peripheral vascular disorders, anxiety disorders, adjustment disorders, allergies, chronic pain, cognitive impairment, depressive disorders, drug related disorders, irritable bowel syndrome, low back pain, migraine, myositis myalgia and fibromyalgia, pelvic and peritoneal pain, post-traumatic stress disorder, reactions to severe stress, sleep disorders, and somatoform disorders.

The cross-validated area under the receiver operating characteristic curve (AUC) in the training set was 0.88. When applied to all current eligible KPNC members as an internal validation set (Fig 1), the AUC was 0.845 with a 95% confidence interval of 0.8396 - 0.8504. The Brier score for the internal validation data, measured comparing predicted probabilities to the actual outcome values, was 0.011. In the training data, using the rule of declaring a positive case if the predicted probability was >0.1794 (to maximize the F1-score, a commonly-used thresholding metric based on a weighted average of precision and recall), the sensitivity of the model was 0.96 and the specificity was 0.42.

Instead of using a numeric cutoff to determine who had a higher probability of ME/CFS, we took the largest predicted probabilities in each race-sex category as outlined in the table below, based on the race and sex distribution in the larger Kaiser Permanente eligible population (S3 Table, column 2; Race & ethnicity, Asian 578,527 (21%), Black 209,281 (7.6%), Latino/Hispanic 436,448 (16%), White 1,340,164 (49%), Other/Unknown 180,954 (6.6%); Sex, Female 1,460,950 (53%), Male 1,284,132 (47%), Other/Unknown 292 (<0.1)):

|  | Asian (20%) | | Black (10%) | | Latino/Hispanic (16%) | | White (50%) | | Other/Unknown* (4%) | | Total |
| --- | --- | --- | --- | --- | --- | --- | --- | --- | --- | --- | --- |
|  | Male | Female | Male | Female | Male | Female | Male | Female | Male | Female |  |
| Number of top predicted probabilities sampled from each category | 3,000 | 3,000 | 1,500 | 1,500 | 2,400 | 2,400 | 7,500 | 7,500 | 600 | 600 | 30,000 |

*Including Native American, Alaska Native, Hawaiian/Pacific Islander, Multiracial, and Unknown race This is due to the fact that the sample would have otherwise been largely white and female.
