# Supplementary material for: Impact of COVID-19 on myalgic encephalomyelitis/chronic fatigue syndrome-like illness prevalence: A cross-sectional survey
Source: PLoS One. 2024 Sep 18;19(9):e0309810. doi: 10.1371/journal.pone.0309810 (PMC11410243; doi:10.1371/journal.pone.0309810)
Supplement: S3 Appendix — (DOCX) [file pone.0309810.s010.docx]

**S3 Appendix. Targeted maximum likelihood estimation description.**

Targeted maximum likelihood estimation (TMLE) is a general framework for constructing asymptotically linear substitution estimators of statistical parameters. Implementation for TMLE will vary by parameter, but in our case, it builds the estimator by leveraging estimates of regressions for an outcome and propensity scores for an exposure. TMLE is “double-robust'' in that it will be consistent if either the outcome regression or propensity score are estimated consistently, and if both are estimated consistently and at appropriate convergence rates, it will achieve the non-parametric efficiency bound, providing the smallest asymptotic variance among a wide class of estimators.

For estimation of the outcome regressions and propensity scores, we use the ensemble machine learning method Super Learner [28] with a diverse set of candidate learners fit via cross-validation, allowing for flexible relationships between variables. Using the ensemble allows us to avoid relying on (possibly mis-specified) parametric models and leverage advances in machine learning. Our Super Learner library included generalized linear models, LASSO, multivariate adaptive regression splines, random forests, gradient boosted trees, and a simple mean.
